# The choice of mitral valve surgery type and mid-term outcomes in patients up to 70 years of age: Results of the AUTHEARTVISIT study

**DOI:** 10.1016/j.xjon.2026.101741

**Published:** 2026-03-27

**Authors:** Johann Auer, Alissa Florian, Christine Wagenlechner, Berthold Reichardt, Ralph Wendt, Michael Mildner, Julia Mascherbauer, Daniel Zimpfer, Hendrik Jan Ankersmit, Alexandra Graf

**Affiliations:** a Department of Internal Medicine I with Cardiology and Intensive Care, St Josef Hospital Braunau, Braunau am Inn, Austria; b Department of Cardiac and Thoracic Aortic Surgery, Medical University of Vienna, Vienna, Austria; c Center for Medical Data Science, Medical University of Vienna, Vienna, Austria; d Department of Thoracic Surgery, Medical University of Vienna, Vienna, Austria; e Austrian Social Health Insurance Fund, Eisenstadt, Austria; f Department of Nephrology, St Georg Hospital, Leipzig, Germany; g Department of Dermatology, Medical University of Vienna, Vienna, Austria; h Department of Internal Medicine 3, University Hospital St Poelten, St Poelten, Austria; i Karl Landsteiner University of Health Sciences, Krems an der Donau, Austria; j Laboratory for Cardiac and Thoracic Diagnosis, Regeneration and Applied Immunology, Vienna, Austria

**Keywords:** bioprosthesis, mechanical replacement, mitral valve, repair

## Abstract

**Objective:**

Mitral valve replacement is indicated when mitral valve repair is not feasible. Prosthesis choice remains controversial, especially in patients aged less than 70 years, and despite existing guidelines, all too often even in those aged less than 65 years. This study investigated long-term survival and adverse outcomes after mechanical mitral valve replacement versus biological mitral valve replacement in a nationwide Austrian cohort, also including mitral valve repair as the control group.

**Methods:**

We performed a retrospective cohort study using Austrian insurance data. It included 3520 patients aged 70 years or less who underwent mitral heart valve surgery between January 1, 2010, and December 31, 2020. The primary end point was all-cause death; secondary end points included time to major adverse cardiac and cerebrovascular events, reoperation, myocardial infarction, heart failure, stroke, intracerebral hemorrhage, and bleeding other than intracerebral hemorrhage. These were analyzed via time-to-event analysis, including Cox proportional hazards regression and cause-specific hazards with inverse probability of treatment weighting, adjusting for demographic and clinically relevant confounders.

**Results:**

Among 3520 patients, 413 underwent mechanical mitral valve replacement, 487 underwent biological mitral valve replacement, and 2620 underwent mitral valve repair. Estimated 10-year survival was 71.6% after mechanical mitral valve replacement, 63.2% after biological mitral valve replacement, and 75.9% after mitral valve repair. Compared with mechanical mitral valve replacement, biological mitral valve replacement was associated with a higher risk of death beyond 4 years (adjusted hazard ratio, 1.94, 95% CI, 1.11-3.39, *P* = .02) and reduced reoperation-free survival (adjusted hazard ratio, 2.10, 95% CI, 1.24-3.56, *P* = .006). Early outcomes showed no significant differences.

**Conclusions:**

In patients aged 70 years or less undergoing mitral valve replacement, mechanical mitral valve replacement provides superior long-term survival compared with biological mitral valve replacement. These findings may support mechanical valves as the preferred option in patients aged less than 70 years.

**Figure:**
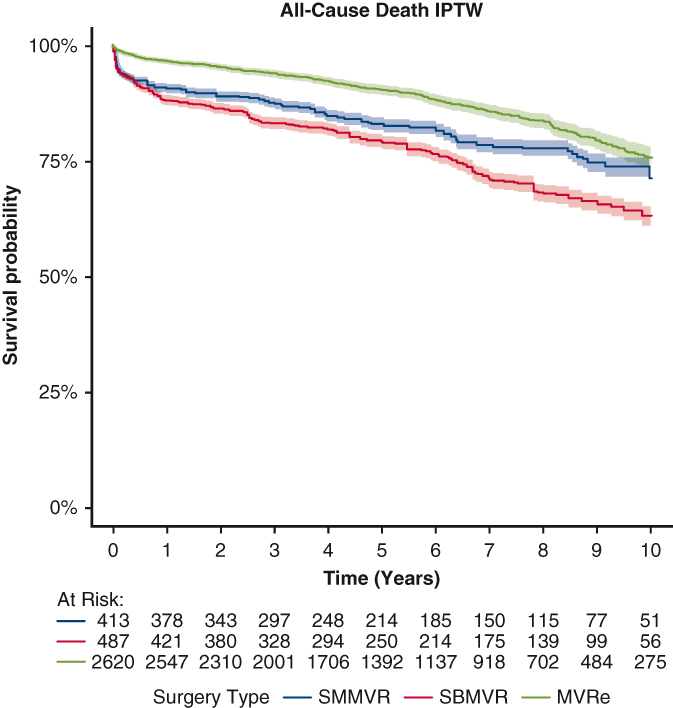
Survival after MVRe, SBMVR, and SMMVR.

**Central Message**

For mitral valve repair, the highest long-term survival was observed, with mechanical replacement slightly lower and bioprosthetic replacement showing the lowest 10-year survival for patients aged less than 70 years.

**Perspective**

This nationwide analysis observed that in patients aged 70 years or less, mechanical MVR offers superior long-term survival and durability compared with bioprostheses, with differences emerging beyond 4 years. These results may support choosing mechanical valves in patients aged 70 years or less when repair is not feasible and highlight the need for careful prosthesis selection to optimize outcomes.

Valvular heart disease is a major global health challenge affecting millions of patients, with increasing prevalence due to aging populations.^1^^,^^2^ For mitral valve disease, mitral valve reconstruction (MVRe) is preferred, but when unfeasible, mitral valve replacement (MVR) using biological or mechanical prostheses is chosen.^3^ Current American guidelines recommend mechanical mitral valve replacement (SMMVR) for patients aged less than 65 years and biological mitral valve replacement (SBMVR) for those aged 65 years or more,^4^ whereas European guidelines^5^ suggest SMMVR for those aged less than 65 years and SBMVR for those aged 70 years or more. Decision-making for the intermediate age group remains controversial, and despite those guidelines, biological prostheses are still implanted far too often in individuals aged less than 65 years.

Data from trials, studies, and meta-analyses show mechanical prostheses provide better long-term survival and durability.^6^, ^7^, ^8^, ^9^, ^10^, ^11^, ^12^, ^13^, ^14^, ^15^, ^16^ The only randomized controlled trial showed more prostheses-related deaths and valve failure after SBMVR, with 95% of patients aged 70 years or less.^6^ Studies confirmed lower mortality in younger patients^7^, ^8^, ^9^, ^10^ and lower reoperation rates^7^, ^8^, ^9^^,^^11^ after SMMVR, although bleeding was higher.^10^

Meta-analyses demonstrated reduced mortality^12^^,^^13^ and reoperation risk in patients aged less than 70 years^13^ and showed reduced durability of biological prostheses in the mitral position.^14^^,^^15^ Although bleeding remains a concern with mechanical prostheses, the reduced durability of biological prostheses makes them less suitable for patients with substantial life expectancy.

Although extensive data exist for aortic valve replacement, mitral valve data are limited. AUTHEARTVISIT database studies showed survival advantages with mechanical aortic valve replacement.^16^, ^17^, ^18^ This study evaluates outcomes after SMMVR, SBMVR, and MVRe in patients aged 70 years or less.

## Patients and Methods

### Study Design

This nationwide, registry-based cohort study using insurance data (AUTHEART VISIT study) was approved by the Ethics Committee of Lower Austria (GS1-EK-4/722-2021, approval date May 6, 2021), registered at clinicaltrials.gov (NCT07168889) and performed in compliance with the Declaration of Helsinki. The study data were retrospectively received from the Austrian Health Insurance Funds, with informed consent waived because of anonymized data. In Austria, health services access is regulated by social insurance law, with most of the population enrolled in public health insurance.

For these analyses, data were obtained for patients in the Austrian Health Care System who underwent surgical MVR using a mechanical prosthesis (SMMVR, MEL code DB102), a biological prosthesis (SBMVR, MEL codes DB090 and DB100), or MVRe (MEL code DB040) from January 1, 2010, to December 31, 2020, and were aged 70 years or less.

Patients with concomitant aortic or pulmonary valve procedures, with other major cardiac or noncardiac interventions (Online Data Supplement), aged more than 70 years, having both SMMVR and SBMVR at index date, and with transcatheter edge-to-edge repair (XN050 MEL Code) or transcatheter mitral valve implantation (XN055 MEL Code) were excluded. Patients with implausible data entries were also excluded (Online Data Supplement).

### Outcomes

Outcomes were defined using billing information (MEL codes) and diagnoses (International Classification of Diseases, 10th Revision codes) from index surgery date to study end (Online Data Supplement). The primary end point was all-cause death from index operation to death date or censoring. Reoperation was defined as the first event after index operation with MEL codes as in Online Data Supplement. Secondary outcomes myocardial infarction, heart failure, embolic stroke or intracerebral hemorrhage (ICH), and bleeding other than ICH were based on International Classification of Diseases, 10^th^ Revision codes (Online Data Supplement). Major adverse cardiac and cerebrovascular events (MACCE) were defined as the first event after index operation, including myocardial infarction, heart failure, embolic stroke or ICH, reoperation, or death.

### Statistical Analysis

Categorical variables are shown as counts and percentages, and continuous variables are shown as medians with first and third quartiles. Standardized mean differences were calculated for group comparisons to evaluate imbalance in comorbidities among groups (SMMVR, SBMVR, MVRe).^19^

The association between surgery type and time until death was evaluated using an inverse probability of treatment weighting (IPTW) multivariable Cox proportional hazards model, accounting for surgery type groups (Online Data Supplement), age, sex, combination surgery, and diagnosis groups: infectious diseases, diabetes mellitus, adiposity, hyperlipidemia, hyperuricemia/gout, valvular, rhythmological, and other cardiomyopathies (CMPs), atherosclerosis, pulmonary disease, stomach and duodenal ulcers and inflammation, intestinal diseases, liver diseases, kidney disease, ischemic CMPs, and malignant diseases (Online Data Supplement). The Cox model was calculated with time-dependent coefficients for intervals 0 to 2, 2 to 4, and more than 4 years to account for the increasing separation of Kaplan–Meier curves between groups. To account for group imbalances, IPTW was performed.^20^ Weighting in the Cox model used an inverse propensity score calculated via multinomial logistic regression including age, sex, and listed diagnosis groups.^21^ IPTW Kaplan–Meier curves used the Aalen-Johanson estimator. Weighted and unweighted Kaplan–Meier curves were plotted showing patients at risk, censored patients, and events yearly up to 10-year follow-up. Survival probabilities with 95% CIs were estimated using the Kaplan–Meier method. Proportional hazard assumption used Schönfeld residuals, and collinearity used variance inflation factors. Cox model results are presented as hazard ratios (HRs) with 95% CIs and *P* values, comparing SBMVR versus SMMVR, SBMVR versus MVRe, and SMMVR versus MVRe. An HR less than 1 indicates an increased probability of the corresponding event in the reference group. Follow-up times used reverse Kaplan–Meier method. Because of the small sample size, the subgroups of patients aged less than 65 years and 65 to 70 years were only presented using unweighted Kaplan–Meier curves.

The combined secondary outcomes of MACCE were analyzed similar to the primary end point all-cause death. Time to reoperation, myocardial infarction, heart failure, stroke or ICH, and bleeding other than ICH were investigated between groups using an ITPW cause-specific proportional hazard regression accounting for surgery type groups, age, sex, combination-surgery (yes/no), and diagnosis groups. The cause-specific proportional hazard model was calculated with time-dependent coefficients of surgery type for intervals 0 to 2, 2 to 4, and more than 4 years. IPTW cumulative incidence curves were drawn using the Aalen-Johanson estimator, and unweighted curves are shown. For heart failure analyses, patients with preindex operation heart failure were excluded to evaluate new diagnoses. Analyses were performed in R (Version 4.5.1). The definition of confounders and outcomes aligns with other AUTHEARTVISIT study analyses.^16^, ^17^, ^18^^,^^22^

## Results

### Study Population and Patient Characteristics

**Figure 1:**
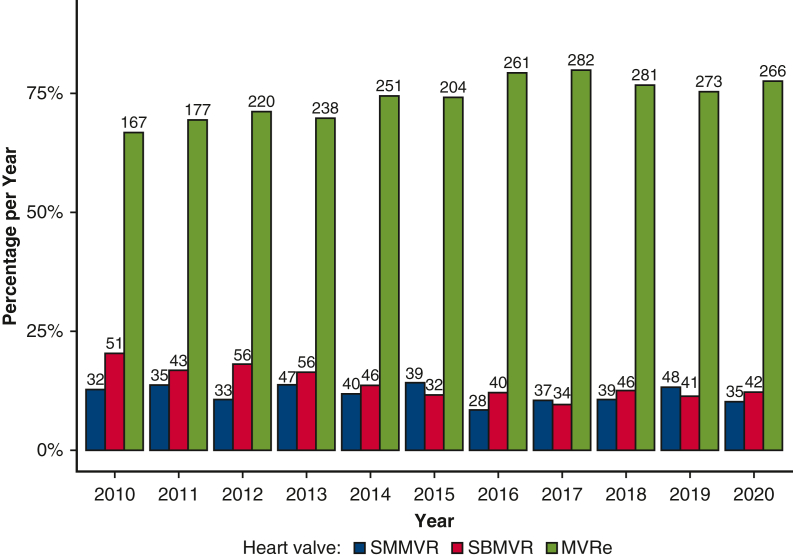
Percentages per year (y-axis) and absolute numbers (above bars) of patients receiving SMMVR, SBMVR, and MVRe. *SMMVR*, Mechanical mitral valve replacement; *SBMVR*, biological mitral valve replacement; *MVRe*, mitral valve reconstruction.

From January 1, 2010, to December 31, 2020, 3520 patients aged 70 years or less underwent mitral valve surgery: 413 SMMVR, 487 SBMVR, and 2620 MVRe. MVRe was most common over the years, whereas SMMVR and SBMVR were performed similarly (Figure 1). SBMVR patients were older with higher rates of infectious diseases, diabetes, adiposity, atherosclerosis, pulmonary, intestinal, liver, kidney diseases, and ischemic CMP compared with MVRe (standardized mean differences >0.1) (Table 1). SBMVR patients showed a higher incidence of infectious diseases, diabetes, atherosclerosis, stomach/duodenal conditions, kidney disease, and ischemic CMPs versus SMMVR (Table 1). MVRe had fewer female patients than other groups. For medication details, see Online Data Supplement. IPTW was performed to address group imbalance. Median follow-up was 6.59 years for SMMVR, 7.44 years for SBMVR, and 5.83 years for MVRe.

**Table 1:** Characteristics and preexisting medical diagnoses at time of index operation and standardized mean differences for description of balance between groups

Variables	All data			SMDs		
	SMMVR (n = 413)	SBMVR (n = 487)	MVRe (n = 2620)	SMMVR vs SBMVR	SMMVR vs MVRe	SBMVR vs MVRe
Median age, y (IQR)	56 (49-61)	65 (58-68)	60 (53-66)	−0.815	−0.354	0.439
Sex, female	196 (47.5%)	238 (48.9%)	790 (30.2%)	0.028	−0.361	−0.390
Infectious diseases	23 (5.6%)	42 (8.6%)	92 (3.5%)	0.119	−0.099	−0.215
Diabetes mellitus	46 (11.1%)	76 (15.6%)	167 (6.4%)	0.132	−0.169	−0.298
Adiposity	26 (6.3%)	37 (7.6%)	113 (4.3%)	0.051	−0.089	−0.139
Hyperlipidemia	66 (16.0%)	89 (18.3%)	444 (17.0%)	0.061	0.026	−0.035
Hyperuricemia/gout	10 (2.4%)	12 (2.5%)	63 (2.4%)	0.003	−0.001	−0.004
Valvular, rhythmological, and other CMPs	319 (77.2%)	385 (79.1%)	2136 (81.5%)	0.044	0.106	0.062
Atherosclerosis	6 (1.5%)	15 (3.1%)	38 (1.5%)	0.110	0.000	−0.110
Pulmonary disease	19 (4.6%)	27 (5.5%)	88 (3.4%)	0.043	−0.064	−0.106
Stomach and duodenal ulcers and inflammation	18 (4.4%)	34 (7.0%)	94 (3.6%)	0.114	−0.039	−0.152
Intestinal diseases	4 (1.0%)	8 (1.6%)	27 (1.0%)	0.059	0.006	−0.053
Liver diseases	17 (4.1%)	25 (5.1%)	71 (2.7%)	0.048	−0.078	−0.125
Kidney disease	29 (7.0%)	74 (15.2%)	169 (6.5%)	0.262	−0.023	−0.284
Ischemic CMPs	112 (27.1%)	187 (38.4%)	817 (31.2%)	0.242	0.090	−0.152
Malignant diseases	5 (1.2%)	8 (1.6%)	64 (2.4%)	0.036	0.092	0.057

Unless otherwise indicated, values are given as the number of patients with a diagnosis and corresponding percentage of total patients in the treatment group. *SMD*, Standardized mean difference; *SMMVR*, mechanical mitral valve replacement; *SBMVR*, biological mitral valve replacement; *MVRe*, mitral valve reconstruction; *IQR*, interquartile range; *CMP*, cardiomyopathy.

### Primary Outcome: All-Cause Death

**Figure 2:**
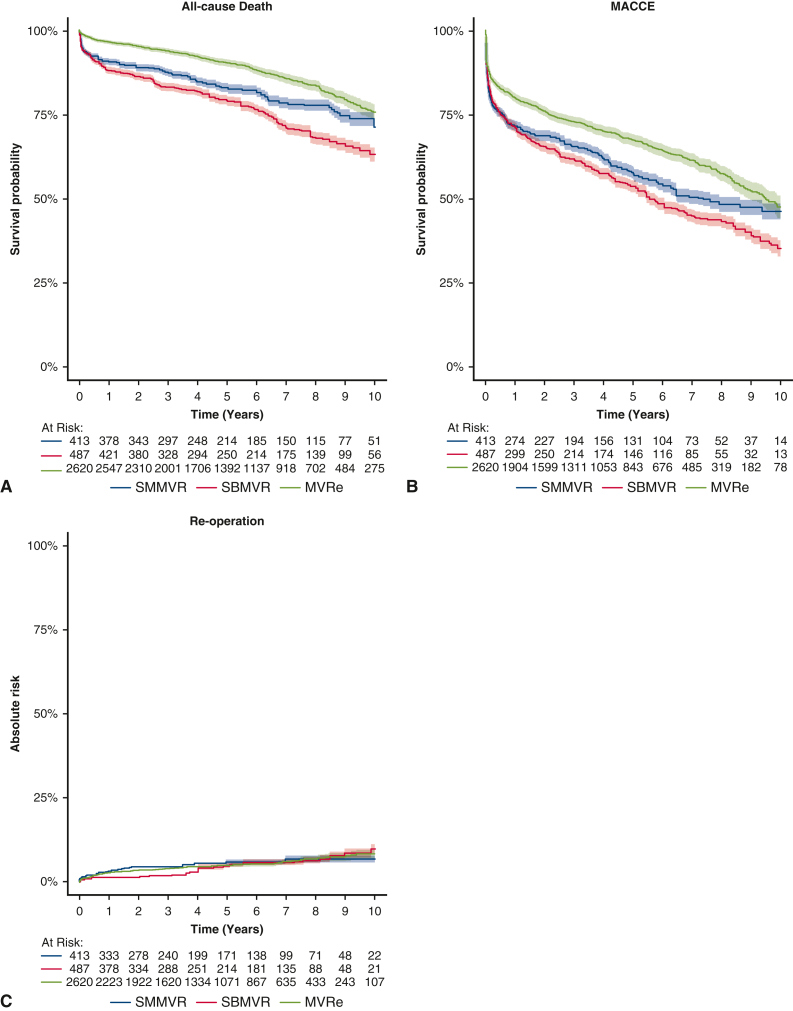
IPTW Kaplan–Meier curves and corresponding 95% CIs for all-cause death (A), MACCE (B), and weighted cumulative incidence curves for reoperation (C). Patients at risk are given below the curves. *SMMVR*, Mechanical mitral valve replacement; *SBMVR*, biological mitral valve replacement; *MVRe*, mitral valve reconstruction.

**Figure 3:**
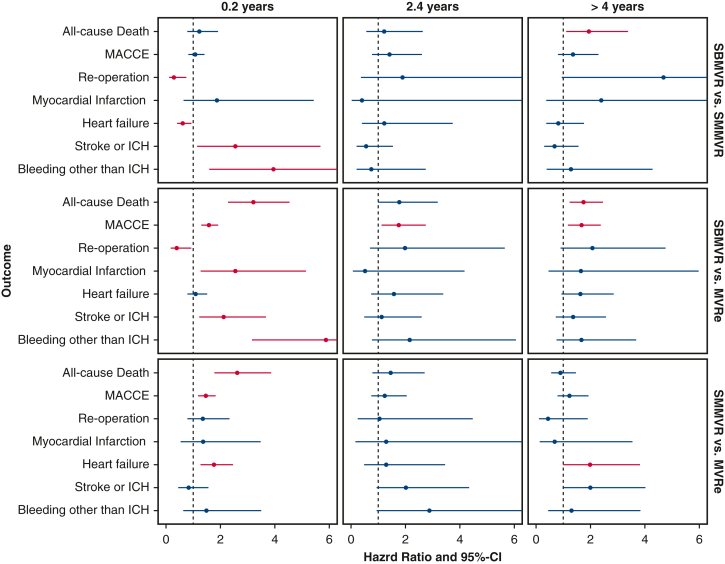
HRs and corresponding 95% CIs from multivariable IPTW Cox regression models for all outcomes for different time periods (first column: 0-2 years, second column: 2-4 years, third column: more than 4 years after index surgery) and all group comparisons (first row: SBMVR vs MVRe, second row: SMMVR vs SBMVR, third row: SMMVR vs MVRe). Significant differences between groups are marked in red. HRs are presented as SBMVR versus SMMVR (reference), SBMVR versus MVRe (reference), and SMMVR versus MVRe (reference); thus, an HR less than 1 indicates an increased probability of the corresponding event in the reference group. *SBMVR*, Biological mitral valve replacement; *MVRe*, mitral valve reconstruction; *SMMVR*, mechanical mitral valve replacement; *MACCE*, major adverse cardiac and cerebrovascular events; *ICH*, intracerebral hemorrhage.

Although a higher risk of all-cause death was observed for SBMVR (Figures 2, *A* and Online Data Supplement), no significant difference was found between SMMVR and SBMVR up to 4 years (0-2 years: adjusted HR, 1.226; 95% CI, 0.783-1.920, *P* = .374, 2-4 years: adjusted HR, 1.217; 95% CI, 0.561-2.638, *P* = .619, Figures 2, *A* and 3). However, SBMVR showed a higher risk more than 4 years after surgery (adjusted HR, 1.941; 95% CI, 1.112-3.388, *P* = .020, Figures 2, *A* and 3). Ten-year survivals were 71.61 (95% CI, 69.26-73.96) for SMMVR, 63.18 (95% CI, 61.08-65.29) for SBMVR, and 75.87 (95% CI, 73.49-78.25) for MVRe (Online Data Supplement). In patients aged 65 to 70 years, 10-year survivals were 61.07 (95% CI, 42.30-79.83) for SMMVR, 57.59 (95% CI, 49.70-65.48) for SBMVR, and 66.83 (95% CI, 61.49-72.17) for MVRe (Online Data Supplement). However, it should be noted that the number of mechanical valves in the 65- to 70-year age group was only 50, meaning the comparison may not be robust. Furthermore, 238 bioprosthetic valves were used in patients aged less than 65 years, which does not align with current guideline recommendations.

SBMVR showed increased death risk compared with MVRe across time intervals (0-2 years: HR, 3.211; 95% CI, 2.272-4.540, *P* < .001, 2-4 years: HR, 1.774; 95% CI, 0.986-3.193, *P* = .056, >4 years: HR, 1.744; 95% CI, 1.234-2.465, *P* = .002, Figures 2, *A* and 3). For SMMVR versus MVRe, a significant difference was only found in the first 2 years (0-2 years: HR, 2.620; 95% CI, 1.774-3.870, *P* < .001, 2-4 years: HR, 1.458; 95% CI, 0.785-2.708, *P* = .233, >4 years: HR, 0.898; 95% CI, 0.550-1.467, *P* = .668, Figures 2, *A* and 3). Because of the small sample sizes in SMMVR and SBMVR groups, these results may be seen as exploratory (Online Data Supplement).

### Secondary Outcomes

#### Major adverse cardiac and cerebrovascular events

No statistically significant difference in the risk of MACCE was found between SMMVR and SBMVR (Online Data Supplement). MVRe showed a significant smaller risk for MACCE compared with SBMVR in all time intervals (0-2 years: HR, 1.577; 95% CI, 1.295-1.920, *P* < .001, 2-4 years: HR, 1.756; 95% CI, 1.120-2.753, *P* = .014, >4 years: HR, 1.673; 95% CI, 1.175-2.382, *P* = .004, Figures 2, *B* and 3). Furthermore, MVRe showed a significant smaller risk for MACCE compared with SMMVR in the first 2 years after index surgery (HR, 1.466; 95% CI, 1.177-1.826, *P* = .001, Figures 2, *B* and 3), but not after 2 years.

#### Reoperation

SBMVR showed a lower reoperation risk in the first 2 years versus SMMVR (HR, 0.291; 95% CI, 0.113-0.749, *P* = .01, Figures 2, *D* and 3, Online Data Supplement), with trend reversal later, although not significant. A similar trend was found versus MVRe. No significant difference existed between SMMVR and MVRe. Note that death risk 10 years after reoperation was observed to be 59.8% (95% CI, 40.6-78.9) over all groups (Online Data Supplement).

#### Myocardial infarction

SBMVR showed a higher myocardial infarction risk in the first 2 years versus MVRe (HR, 2.556; 95% CI, 1.269-5.149, *P* = .009, Figure 3, Online Data Supplement), with no significant difference later or between other groups.

#### Heart failure

For SBMVR, a statistically significant trend for a lower risk of heart failure was observed in the first 2 years after index surgery compared with SMMVR (HR, 0.617; 95% CI, 0.406-0.937, *P* = .023, Figure 3, Online Data Supplement), but the trend was not significant after 2 years. A significantly larger risk for heart failure was found for SMMVR compared with MVRe in the first 2 years after index surgery (HR, 1.766; 95% CI, 1.264-2.468, *P* = .001) and more than 4 years after index surgery (HR, 1.987; 95% CI, 1.033-3.822, *P* = .04). No statistically significant difference was observed between SBMVR and MVRe.

#### Embolic Stroke and Intracerebral Hemorrhage

A significantly higher risk for embolic stroke was found for SBMVR in the first 2 years after index surgery compared with SMMVR (HR, 2.547; 95% CI, 1.142-5.680, *P* = .022, Figure 3, Online Data Supplement), the trend reversing but not being significant in later years. A significant higher risk for embolic stroke was also found for SBMVR in the first 2 years after index surgery compared with MVRe (HR, 2.121; 95% CI, 1.223-3.681, *P* = .007) but not in later years. No statistically significant difference was observed between SMMVR and MVRe.

#### Bleeding other than intracerebral hemorrhage

A significantly higher risk for bleeding other than ICH was found for SBMVR in the first 2 years after index surgery compared with SMMVR (HR, 3.948; 95% CI, 1.587-9.822, *P* = .003; Figure 3, Online Data Supplement), but the trend was not significant after 2 years. A similar trend was found for SBMVR compared with MVRe. No statistically significant risk for bleeding other than ICH was found for SMMVR compared with MVRe. However, because of the small sample sizes and the low number of events, results may be interpreted with caution.

## Discussion

In this nationwide, registry-based analysis of patients aged 70 years or less undergoing mitral valve surgery, mechanical prostheses showed superior long-term survival compared with bioprostheses, with differences becoming significant after 4 years. Our findings support existing literature favoring mechanical valves in the mitral position for patients aged less than 70 years. The Veterans Affairs trial^6^ showed no significant difference in death after 15 years between SMMVR and SBMVR (81% ± 4% vs 79% ± 4%; *P* = .3), whereas prostheses-related deaths were more common after SBMVR (57%) than SMMVR (44%). Primary valve failure was significantly higher after SBMVR (44% ± 8% vs 5% ± 4%; *P* = .0002). Goldstone and colleagues^7^ showed SBMVR had higher mortality versus SMMVR in patients aged 40 to 49 years (44.1% vs 27.1%; HR 1.88; 95% CI, 1.35-2.63; *P* < .001) and 50 to 69 years (50.0% vs 45.3%; HR, 1.16; 95% CI, 1.04-1.30; *P* = .01), plus higher reoperation rates. Rokui and colleagues^8^ found better 10-year survival with SMMVR versus SBMVR in patients aged less than 65 years (78.2% vs 69.8%, *P* < .029) and higher freedom from reintervention (96.2% vs 81.3%, *P* < .001). Similar to our results, Cetinkaya and colleagues^10^ found SBMVR showed higher survival initially, shifting to favor SMMVR after 3 years, with 10-year rates of 62.4% versus 77.1% for SBMVR versus SMMVR (*P* = .769, HR, 0.833; 95% CI, 0.430-1.615).

Our results are in line with prior meta-analyses, such as by Yu and colleagues,^13^ who demonstrated reduced longtime mortality after SMMVR in unadjusted (HR, 0.77; 95% CI, 0.70-0.84; *P* < .0001) and adjusted groups (HR, 0.84; 95% CI, 0.77-0.91; *P* < .0001), and lower reoperation risk in both adjusted (HR, 0.34; 95% CI, 0.23-0.50; *P* < .00001) and unadjusted groups (HR, 0.31; 95% CI, 0.18-0.53; *P* < .0001). Koulouroudias and colleagues^15^ analyzed 20 SBMVR studies, finding 15-year overall survival of 40% (95% CI, 38-42), freedom from reoperation of 79% (95% CI, 76-82), and freedom from structural valve deterioration (SVD) of 64% (95% CI, 58-70). At 15 years, freedom from SVD was 46% (95% CI, 32-67) in patients aged 18 to 59 years, 64% (95% CI, 50-83) in patients aged 60 to 69 years, and 93% (95% CI, 87-100) at 10 to 25 years in patients aged 70+ years. The HR was 6.6 (95% CI, 2.5-17, *P* < .0001) for 18 to 59 years versus 70+ years and 2.5 (95% CI, 0.9-6.9, *P* < .0001) for 60 to 69 years versus 70+ years, showing faster SVD in younger patients.

These observations on SVD align with studies on aortic bioprostheses. Percy and colleagues^23^ reported that more than 40% of patients aged less than 65 years developed subclinical SVD, with 21.5% progressing to clinical SVD within 11 months. Salaun and colleagues^24^ showed leaflet calcifications in 25.6% of patients and valve deterioration in 38% after 6.7 months. Our group has demonstrated that bovine or porcine valve implantation triggers an alpha-Gal–specific immune response linked to SVD.^25^ Another group showed that biological prosthesis implantation induces anti-alpha-Gal and anti-Neu5Gc expression with increased complement deposition.^26^ These findings show that xenogenic valve implantation triggers immune responses, contributing to SVD progression. Given these data, SMMVR may be better for patients aged 70 years or less. Although studies show bleeding and thromboembolism remain more common after SMMVR than SBMVR, we found no statistically significant worse outcome, but the results may be limited by sample size.

Although anticoagulation complications such as bleeding and thromboembolism remain important after SMMVR, they must be viewed against mechanical prostheses' durability advantage. Because vitamin K antagonists remain the only safe anticoagulation strategy, management is challenging. SVD remains unavoidable and may lead to more reoperations in SBMVR patients.

Although our previous work showed survival benefits for mechanical prostheses in the aortic position,^16^, ^17^, ^18^ this analysis suggests this advantage extends to the mitral position, indicating mechanical prostheses may be preferred for patients aged 70 years or less.

### Limitations

We observed real-world data from Austrian insurance funds, acknowledging observational research limitations. Data were collected from all patients who underwent SMVR from January 1, 2010, to December 31, 2020, with approximately 98% of Austria's population registered in the healthcare system. Because our retrospective data lacked the rigor of prospective randomized trials, we used IPTW to account for group differences, reducing potential bias that MVRe or SMMVR might be performed more often in younger, healthier individuals. IPTW was chosen over propensity score matching to preserve the full sample and avoid loss of precision due to unmatched subjects, which may lead to an overly small sample size. Because of administrative data, we did not have to deal with missing values; however, data are derived from billing records that depend on accurate nationwide coding, which we cannot retrospectively verify, potentially introducing bias compared with prospective databases. We cannot determine the pathology that led to the surgeries or medical reasons for death or reoperation, preventing exclusion of unrelated outcomes. However, our outcome measures involved hospital events likely to be accurately reported for billing. Sample sizes in SMMVR and SBMVR groups and event rates for secondary outcomes were low, requiring cautious interpretation.

## Conclusions

In this nationwide cohort of patients aged 70 years or younger undergoing mitral valve surgery, mitral valve repair achieved the best overall survival. When replacement was necessary, mechanical prostheses were associated with better long-term survival and durability than bioprostheses, supporting their preferential use in younger patients when repair is not feasible.

## Data Availability

Due to data protection, the datasets presented in this article are not readily available. Data that support the findings of this study are available upon reasonable request from the corresponding authors.

## Conflict of Interest Statement

The authors reported no conflicts of interest.

The *Journal* policy requires editors and reviewers to disclose conflicts of interest and to decline handling or reviewing manuscripts for which they may have a conflict of interest. The editors and reviewers of this article have no conflicts of interest.
