# Supplementary material for: The choice of mitral valve surgery type and mid-term outcomes in patients up to 70 years of age: Results of the AUTHEARTVISIT study
Source: JTCVS Open. 2026 Mar 27;31:101741. doi: 10.1016/j.xjon.2026.101741 (PMC13316340; doi:10.1016/j.xjon.2026.101741)
Supplement: Online Data Supplement [file mmc1.pdf]

Supplement to

The choice of mitral valve surgery type and mid-term outcomes in patients up to 70-years: results of the AUTHEARTVISIT Study

J. Auer[1#], A. Florian[2#], C. Wagenlechner[3,4], B. Reichardt[5], R. Wendt[6], M. Mildner[7], J. Mascherbauer[8,9], D. Zimpfer[2*], HJ Ankersmit[4,10*], A. Graf[3]

[1] Department of Internal Medicine I with Cardiology and Intensive Care, St. Josef Hospital Braunau, Braunau am Inn, Austria

[2] Department of Cardiac and Thoracic Aortic Surgery, Medical University of Vienna, Vienna, Austria

[3] Center for Medical Data Science, Medical University of Vienna, Vienna, Austria

[4] Department of Thoracic Surgery, Medical University of Vienna, Austria

[5] Austrian Social Health Insurance Fund, Eisenstadt, Austria

[6] Department of Nephrology, St. Georg Hospital, Leipzig, Germany

[7] Department of Dermatology, Medical University of Vienna, Austria

[8] Department of Internal Medicine 3, University Hospital St. Poelten, St. Poelten, Austria

[9] Karl Landsteiner University of Health Sciences, Krems an der Donau, Austria

[10] Laboratory for Cardiac and Thoracic Diagnosis, Regeneration and Applied Immunology, Austria

# J. Auer and A. Florian contributed equally to this work

* Corresponding authors:
Univ. Prof. PD Daniel Zimpfer, MBA
Department of Cardiac Surgery
Medical University of Vienna
Währinger Gürtel 18-20
1090 Vienna
Tel. 0043-1-40400- 69660
email: daniel.zimpfer@meduniwien.ac.at

Univ. Prof. Hendrik Jan Ankersmit
Department of Thoracic Surgery
Medical University of Vienna
Währinger Gürtel 18-20
1090 Vienna
Tel. 0043-1-40400-67770
e-mail: hendrik.ankersmit@meduniwien.ac.at
www.applied-immunology.at

1.) General information on the cohort analyzed

This retrospective national registry-based study complied with the Declaration of Helsinki and was approved by the ethics committee of lower Austria (GS1-EK-4/722-2021). The trial was registered with ClinicalTrials.gov (NCT05912660). Study data were generated retrospectively by retrieval from the Austrian Health Insurance Funds. Data on outcomes and potential confounding factors were derived from billing information based on MEL (i.e., Medizinische Einzelleistung, or individual medical procedure) and International Classification of Diseases (ICD) codes available for each patient from one year before surgery up to study cut-off.

Austria's health care system operates as a national framework with broad access to medical care. The access to health services is regulated by social insurance law. All insured individuals have a legal entitlement to services. Austrian social insurance is founded on the principles of solidarity and self-administration, primarily financed through social insurance contributions. Around 98% of the Austrian population is enrolled in the public health insurance system. Therefore, only a small group of privately insured patients covering medical expenses could not be included in the AUTHEART-VISIT-Study. A flow chart of in- and excluded patients is shown in Figure S1

0.1. Inclusion Criteria

For the presented analyses of the AUTHEARTVISIT study, clinical and operative data were obtained for all patients registered in the Austrian Health Care System who underwent surgical mitral valve replacement (SMVR) using a mechanical prosthesis (SMMVR, MEL code DB102 [Replacement of mitral valve with artificial mechanical valve]), biological prosthesis (SBMVR, MEL codes DB090 [Replacement of mitral valve with stentless valve] and DB100 [Replacement of mitral valve with stented valve]) or mitral valve reconstruction (MVRe, MEL code DB040 [Reconstruction of mitral valve]) in Austria from 01.01.2010 to 31.12.2020 and that were aged 70 years or below.

0.2. Exclusion Criteria

Patients having combined surgery of SMMVR and SBMVR at index date were excluded from the analysis.
Patients with clip (MEL-Code XN050 [Implantation of a mitral valve clip]) or TAMV (MEL-Code XN055 [Mitral valve replacement – catheter-assisted, transapical]) were excluded from the analysis.
Patients who had mitral valve surgery combined with concomitant aortic or pulmonary valve procedures, or with other major cardiac or non-cardiac interventions likely to bias outcomes (see Table S1) were excluded from the sample.
Patients aged >70 years were excluded from the data.
Furthermore, patients with implausible data entries as e.g. the death date before index surgery or implausible additional MEL-codes were excluded.

MEL-Code	Description
DB060	Replacement of aortic valve with pulmonary autograft
DA040	Myocardial biopsy
DA050	Systemic thrombolytic therapy for myocardial infarction
DA070	Resection of ventricular aneurysm
DA080	Percutaneous closure of intracardiac septal defects (ASD, VSD)
DA090	Resection of intracavitary cardiac tumor
DA110	Correction of congenital heart defects with cardiopulmonary bypass (isolated ASD, isolated VSD)
DA120	Correction of complex congenital heart defects with cardiopulmonary bypass
DB021	Aortic valve replacement – percutaneous, interventional, TAVR
DB025	Aortic valve replacement – catheter directed, transapikal, TAVR
DB026	Aortic valve replacement – catheter directed, transvalvular, TAVR
DB055	Pulmonary valve reconstruction
DB070	Replacement of aortic valve with stentless valve
DB080	Replacement of aortic valve with stented valve
DB082	Replacement of aortic valve with artificial mechanical valve
DB130	Replacement of pulmonary valve with stentless valve
DC040	Pericardiectomy with cardiopulmonary bypass
DD040	PTCA – Percutaneous transluminal coronary angioplasty
DD050	Coronary stent implantation

DD060	Drug-eluting stent implantation in coronary arteries
DD080	Intracoronary thrombus aspiration
DD090	PTCA with cutting balloon
DD100	Percutaneous transluminal coronary atherectomy
DE132	Transvenous extraction of pacemaker leads
DF060	Pulmonary artery embolectomy with cardiopulmonary bypass
DG010	Catheter angiography – aorta
DG030	Thoracic aortic stent graft implantation
DG070	Reconstruction of ascending aorta without valve replacement/reconstruction
DG080	Reconstruction of ascending aorta without valve replacement under circulatory arrest
DG090	Reconstruction of ascending aorta with aortic valve replacement (per session)
DG100	Reconstruction of ascending aorta with aortic valve replacement under circulatory arrest
DG110	Reconstruction of ascending aorta with aortic valve repair
DG120	Reconstruction of ascending aorta and aortic valve under circulatory arrest
DG130	Partial reconstruction of aortic arch under circulatory arrest
DG140	Total reconstruction of aortic arch under circulatory arrest
DG150	Reconstruction of descending thoracic aorta with vascular graft
DL041	Implantation of paracorporeal univentricular assist device
DL051	Implantation of intracorporeal univentricular assist device
DL052	Implantation of intracorporeal biventricular assist device
DZ060	Suture repair of cardiac injury
DZ070	Heart transplantation
DZ099	Other surgery – heart and adjacent aorta
EA050	Percutaneous transluminal thrombectomy of intracranial vessels
EB010	Catheter angiography – head and neck
EB050	Arterial suture – extracranial vessels of head and neck
EB060	Carotid artery reconstruction (thrombendarterectomy, vascular graft)
EC050	Arterial suture – upper extremity, thorax
EC062	Endarterectomy/thrombendarterectomy – upper extremity
EC070	Surgical revascularization of upper extremity/thoracic arteries
EC080	Subclavian artery transposition
ED058	Percutaneous transluminal recanalization with stent implantation – pelvic arteries
ED070	Arterial suture – abdomen/pelvis
ED080	Surgical revascularization of pelvic artery without graft
EF040	Percutaneous transluminal recanalization with stent implantation – lower extremity
EF050	Arterial suture – lower extremity
EF060	Endarterectomy/thrombendarterectomy – lower extremity
EF090	Surgical revascularization of femoral artery with graft
EF100	Surgical revascularization of femoral artery with prosthetic material
EJ010	Hepatic vein catheterization and indirect portal pressure measurement (without biopsy)
EJ040	Creation of portosystemic shunt
EK030	Endoscopic dissection of perforator veins
EK060	Reconstruction of pelvic or femoral vein
EK079	Other venous surgery
EP020	Percutaneous transluminal embolectomy/thrombectomy or foreign body removal (excl. intracranial vessels)
EP040	Arterial embolectomy or thrombectomy
EP069	Other arterial surgery
EQ010	Venous embolectomy or thrombectomy
FC018	Sentinel lymph node removal
FC080	Radical mediastinal lymphadenectomy
FC169	Other surgery – lymph nodes/lymphatic vessels
FF050	Open splenectomy
FV080	Non-selective plasmapheresis
FV090	Selective plasmapheresis

GE010	Bronchoscopy
GE089	Other surgery – trachea
GF030	Thoracoscopy – diagnostic
GF040	Thoracoscopy – therapeutic
GF050	Thoracotomy – therapeutic
GF070	Wedge resection of lung – open
GF080	Wedge resection of lung – thoracoscopic
GF120	Lobectomy or bilobectomy – open
GG020	Pleural decortication – open
GH010	Resection of mediastinal mass – open
HL020	Liver suturing/hemostasis
HL060	Liver transplantation
HL079	Other liver surgery
HP010	Exploratory laparotomy
HP030	Therapeutic laparotomy– therapeutisch (LE=je Sitzung)
HP050	Re-laparotomy during same hospital stay
HP070	Re-laparotomy with placement/replacement of intra-abdominal vacuum dressing
HZ020	Endoscopic bougienage of GI tract
JE069	Other urethral surgery
JH060	Orchiectomy via scrotal approach
JN040	Cesarean section
LJ059	Other surgery – thorax/thoracic wall
LZ032	Resection/debulking of soft tissue or bone tumors of head, neck, trunk
LZ100	Surgical removal of multiple retained or impacted teeth
NJ090	Fasciotomy – lower extremity
NZ179	Other surgery – musculoskeletal system of lower extremity
QE040	Partial mastectomy without axillary lymph node dissection
QZ109	Other surgery – skin, skin appendages, subcutaneous tissue
XA300	Antithymocyte globulin or antilymphocyte globulin for organ transplantation
XN010	Aortic valve replacement – percutaneous, interventional, TAVR
ZN050	Preoperative tissue marking – ultrasound-guided

Table S1: Observed combined medical devices additional to Mitral valve surgery leading to exclusion of patients from the data set.

Figure S1: Flow-diagram for in- and exclusion of patients

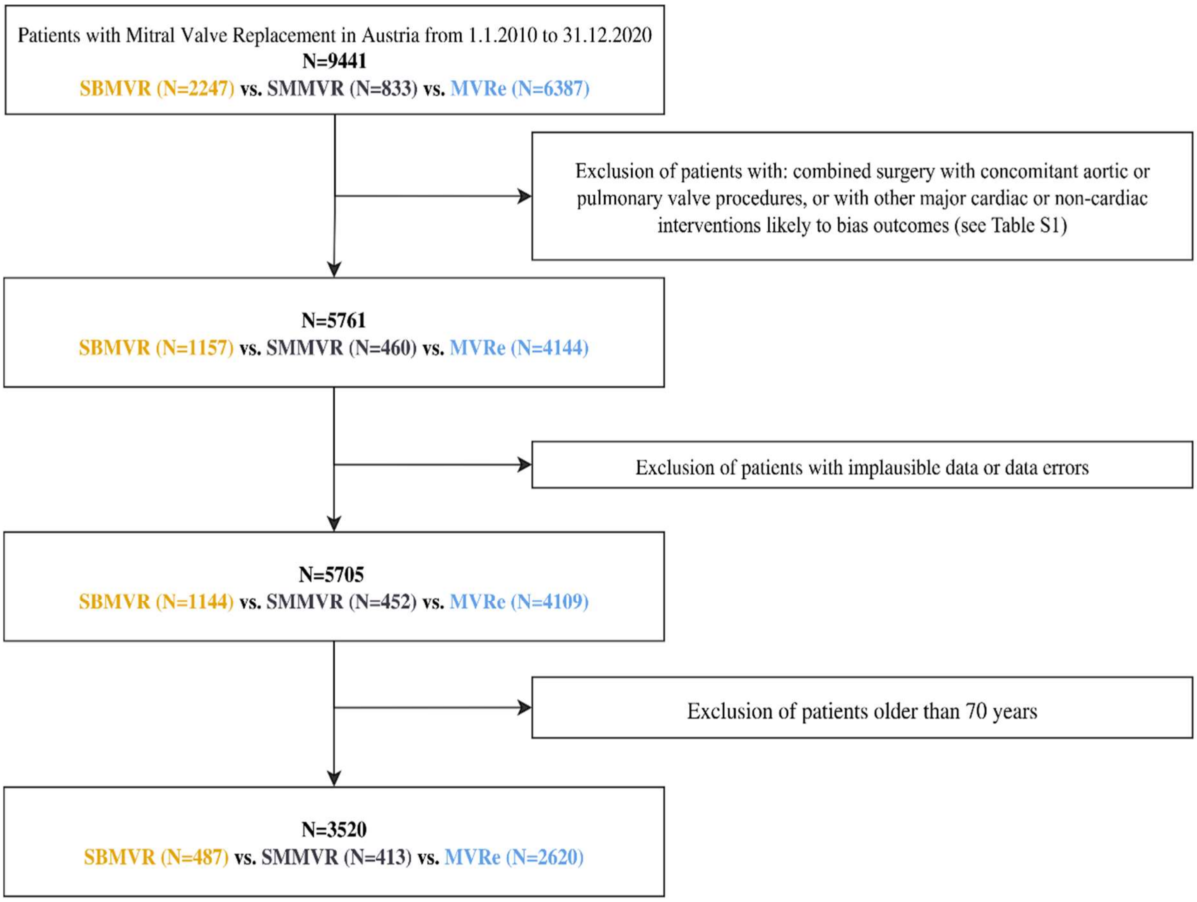

2.) Definition of Outcomes

For each patient, billing information (based on MEL codes from the Austrian insurance carriers) and diagnoses (based on ICD-10 codes) were available from 1 year before the index operation to the end of the study. To evaluate the diagnoses, we used the 10th revision of the International Statistical Classification of Diseases and Related Health Problems (ICD-10 from 2019), which is available at

https://icd.who.int/browse10/2019/en

The corresponding German version is available at

https://www.dimdi.de/static/de/klassifikationen/icd/icd-10-who/kode-suche/htmlamtl2019/.

For each patient, billing information (based on MEL codes) and diagnoses (based on ICD-10 codes) were available from 1 year before the index operation to the end of the study. Death dates were available until the end of the study. To evaluate the different outcomes for each patient, data were scanned for the corresponding codes from the index operation to the end of the study as shown in the following tables. Index operation (Index-OP) was either SMMVR, SBMVR or MVRe as described in the Inclusion Criteria (Section 1.1).

**Outcome**	**Definition**
**Primary**	
All-cause death	All-cause death based on death date (time from Index-OP to death)
**Secondary**	
MACCE	Major adverse cardiac or cerebrovascular event defined as combined endpoint: time from Index-OP to the first event after Index-OP with ICD codes (Table S3) for myocardial infarction, heart failure, embolic stroke or ICH as well as, reoperation, or death based on death date
Reoperation	Based on billing information (MEL code): time from Index-OP to the first event after Index-OP on with MEL code defined as in Table S3
Myocardial infarction	Based on ICD codes: time from the Index-OP to the first event after Index-OP with ICD code for Myocardial infarction defined as in Table S3
Heart failure	Based on ICD codes: time from Index-OP to the first event after Index-OP with ICD code for Heart failure defined as in Table S3
Embolic stroke or intracerebral hemorrhage (ICH)	Based on ICD codes: time from Index-OP to the first event after Index-OP with ICD code for Embolic stroke or ICH defined as in Table S3
Bleeding other than ICH	Based on ICD codes: time from Index-OP to the first event after Index-OP with ICD code for Bleeding other than ICH defined as in Table S3

Table S2: Definitions of outcomes

**Outcome**	**ICD-10 Codes**
Myocardial infarction	I21.0, I21.1, I21.2, I21.3, I21.4, I21.9
Heart failure	I11.0, I13.0, I13.2, I50.0, I50.1, I50.9, I50.11, I50.12, I50.13, I50.14, I50.19
Embolic stroke or intracerebral hemorrhage (ICH)	I63.0, I63.1, I63.2, I63.3, I63.4, I63.5, I63.6, I63.8, I63.9, G45.9, G45.0, G45.1, G45.2, G45.3, G45.4, G45.8, I61.0, I61.1, I61.2, I61.3, I61.4, I61.5, I61.6, I61.8, I61.9, I64
Bleeding other than ICH	I60.0, I60.1, I60.2, I60.3, I60.4, I60.5, I60.6, I60.7, I60.8, I60.9, I85.0, I98.2, I98.3, K25.0, K25.1, K25.2, K25.3, K25.4, K25.5, K25.6, K25.7, K25.9, K26.0, K26.1, K26.2, K26.3, K26.4, K26.5, K26.6, K26.7, K26.9, K27.0, K27.1, K27.2, K27.3, K27.4, K27.5, K27.6, K27.7, K27.9, K28.0, K28.1, K28.2, K28.3, K28.4, K28.5, K28.6, K28.7, K28.9, K29.0, K29.1, K29.2, K29.3, K29.4, K29.5, K29.6, K92.2, N42.1, R04.1, R04.8, R04.9, R58, S06.4, T81.0

Table S3: ICD-10 codes for the definition of outcomes

MEL Code	Description
DB020	Percutaneous implantation of a pulmonary valve
DB025	Aortic valve replacement – catheter directed, transapical, TAVR
DB026	Aortic valve replacement – catheter directed, transvalvular, TAVR
DB030	Reconstruction of the aortic valve
DB040	Reconstruction of the mitral valve
DB050	Reconstruction of the tricuspid valve
DB055	Reconstruction of the pulmonary valve
DB060	Replacement of aortic valve with pulmonary autograft
DB070	Replacement of aortic valve with stentless valve
DB080	Replacement of aortic valve with stented valve
DB082	Replacement of aortic valve with artificial mechanical valve
DB090	Replacement of mitral valve with stentless valve
DB100	Replacement of mitral valve with stented valve
DB102	Replacement of mitral valve with artificial mechanical valve
DB110	Replacement of tricuspid valve with stentless valve
DB120	Replacement of tricuspid valve with stented valve
DB122	Replacement of tricuspid valve with artificial mechanical valve
DB130	Replacement of pulmonary valve with stentless valve
DB140	Replacement of pulmonary valve with stented valve
DB142	Replacement of pulmonary valve with artificial mechanical valve
DB021	Aortic valve replacement – percutaneous, interventional, TAVR
XN010	Aortic valve replacement – percutaneous, interventional, TAVR
XN050	Implantation of a mitral valve clip – percutaneous
XN055	Mitral valve replacement – catheter-assisted, transapical

Table S4: MEL-codes for the definition of the outcome reoperation

3.) **Definitions of Confounders/Comorbidities**

The index operation group group was defined using billing information based on MEL codes as in Table S4.

MEL Code	Description	Group
DB090	Replacement of mitral valve with stentless valve	SBMVR
DB100	Replacement of mitral valve with stented valve	SBMVR
DB102	Replacement of mitral valve with artificial mechanical valve	SMMVR
DB040	Reconstruction of the mitral valve	MVRe

Table S5: Coding for prosthesis variable. SBMVR: biological mitral valve replacement; SMMVR: mechanical mitral valve replacement; MVRe: mitral valve reconstruction

Comorbidities were defined using ICD-10 codes available for each patient up to 1 year before the index operation. Data available 1 year before the index operation were scanned for each patient based on the following ICD-10 codes, categorized for different comorbidities (Table S6). If at least once during the year prior to the index operation an ICD-10 code for a comorbidity was observed as the main or secondary diagnosis, the patient was assumed to suffer from this comorbidity.

Comorbidity	ICD Codes
Infectious diseases	A40.0, A40.1, A40.2, A40.3, A40.8, A40.9, A41.0, A41.1, A41.2, A41.3, A41.4, A415, A41.8, A41.9, A46, A48.3, A48.8, A49.0, A49.1, A49.8, A49.9, A69.2, A69.8, B00.1, B00.2, B00.3, B00.4, B00.5, B00.7, B00.8, B00.9, B02.0, B02.1, B02.2, B02.3, B02.7, B02.8, B02.9, B18.0, B18.00, B18.09, B18.1, B18.10, B18.19, B18.2, B18.8, B189, B200, B20.1, B20.2, B20.3, B20.4, B205, B206, B207, B208, B209, B210, B211, B212, B21.3, B21.7, B21.8, B21.9, B22.0, B22.1, B22.2, B22.7, B24, B33.2, B90.8, B90.9, B95.0, B95.1, B95.2, B95.3, B95.4, B95.5, B95.6, B95.7, B95.8, B97.1, B97.2, B97.3, B99, G04.2, G04.8, G04.9, G05.0, G05.1, G05.2, G05.8, G06.0, G06.1, G06.2, G07, G08, J39.0, J39.1, L00, L01.0, L01.1, L02.0, L02.1, L02.2, L02.3, L02.4, L02.8, L02.9, L03.0, L03.1, L03.2, L03.3, L03.8, L03.9, M00.0, M00.00, M00.01, M00.02, M00.03, M00.04, M00.05, M00.06, M00.07, M0008, M00.09, M00.1, M00.10, M00.11, M00.12, M00.13, M00.14, M00.15, M00.16, M0017, M00.18, M00.19, M00.2, M00.20, M00.21, M00.22, M00.23, M00.24, M00.25, M00.26, M00.27, M00.28, M00.29, M00.8, M00.80, M00.81, M0082, M00.83, M00.84, M00.85, M00.86, M00.87, M00.88, M00.89, M00.9, M0090, M0091, M00.92, M00.93, M00.94, M00.95, M00.96, M00.97, M00.98, M00.99, M01.0, M01.00, M01.01, M01.02, M01.03, M01.04, M01.05, M01.06, M01.07, M01.08, M01.09, M01.1, M01.10, M01.11, M01.12, M01.13, M01.14, M01.15, M01.16, M01.17, M01.18, M01.19, M01.2, M01.20, M0121, M01.22, M01.23, M01.24, M01.25, M01.26, M01.27, M01.28, M01.29, M01.3, M0130, M01.31, M01.32, M01.33, M01.34, M01.35, M01.36, M01.37, M01.38, M01.39, M86.3, M86.30, M86.31, M86.32, M86.33, M86.34, M86.35, M86.36, M86.37, M86.38, M86.39, M86.4, M86.40, M86.41, M86.42, M86.43, M86.44, M86.45, M86.46, M86.47, M86.48, M86.49, M86.5, M86.50, M86.51, M86.52, M86.53, M86.54, M86.55, M86.56, M86.57, M86.58, M86.59, M86.6, M86.60, M86.61, M86.62, M86.63, M86.64, M86.65, M86.66, M86.67, M86.68, M86.69, M86.8, M86.80, M86.81, M86.82, M86.83, M86.84, M86.85, M86.86, M86.87, M86.88, M86.89, M86.9, M86.90, M86.91, M86.92, M86.93, M86.94, M86.95, M86.96, M86.97, M86.98, M86.99
Diabetes mellitus	E10.0, E10.1, E10.2, E10.3, E10.4, E10.5, E10.6, E10.7, E10.8, E10.9, E11.0, E11.1, E11.2, E11.3, E11.4, E11.5, E11.6, E11.7, E11.8, E11.9, E12.0, E12.1, E12.2, E12.3, E12.4, E12.5, E12.6, E12.7, E12.8, E12.9, E13.0, E13.1, E13.2, E13.3, E13.4, E13.5, E13.6, E13.7, E13.8, E13.9, E14.0, E14.1, E14.2, E14.3, E14.4, E14.5, E14.6, E14.7, E14.8, E14.9
Adiposity	E65, E66.0, E66.1, E66.2, E66.8, E66.9
Hyperlipidemia	E78.0, E78.1, E78.2, E78.3, E78.4, E78.5, E78.6, E78.8, E78.9
Hyperuricemia/gout	E79.0, E79.8, M10.0, M10.00, M10.01, M10.02, M10.03, M10.04, M10.05, M10.06, M10.07, M10.08, M10.09
Valvular, rhythmological, and other cardiomyopathies (CMPs)	I01.0, I01.1, I01.2, I01.8, I01.9, I02.0, I02.9, I05.0, I05.1, I05.2, I05.8, I05.9, I06.0, I06.1, I06.2, I06.8, I06.9, I07.0, I07.1, I07.2, I07.8, I07.9, I08.0, I08.1, I08.2, I08.3, I08.8, I08.9, I09.0, I09.1, I09.2, I09.8, I09.9, I10, I11.0, I11.9, I12.0, I12.9, I13.0, I13.1, I13.2, I13.9, I15.0, I15.1, I15.2, I15.8, I15.9, I26.0, I26.9, I27.0, I27.1, I27.2, I27.8, I27.9, I28.0, I28.1, I28.8, I28.9, I30.0, I30.1, I30.8, I30.9, I31.0, I31.1, I31.2, I31.3, I31.8, I31.9, I32.0, I32.1, I32.8, I33.0, I33.9, I34.0, I34.1, I34.2, I34.8, I34.9, I35.0, I35.1, I35.2, I35.8, I35.9, I36.0, I36.1, I36.2, I36.8, I36.9, I37.0, I37.1, I37.2, I37.8, I37.9, I38, I39.0, I39.1, I39.2, I39.3, I39.4, I39.8, I40.0, I40.1, I40.8, I40.9, I41.0, I41.1, I41.2, I41.8, I42.0, I42.1, I42.2, I42.3, I42.4, I42.5, I42.6, I42.7, I42.8, I42.9, I43.0, I43.1, I43.2, I43.8, I44.0, I44.1, I44.2, I44.3, I44.4, I44.5, I44.6, I44.7, I45.0, I45.1, I45.2, I45.3, I45.4, I45.5, I45.6, I45.8, I45.9, I46.0, I46.1, I46.9, I470., I47.1, I47.2, I47.9, I48.0, I48.1, I48.2, I48.3, I48.4, I48.9, I49.0, I49.1, I49.2, I49.3, I49.4, I49.5, I49.8, I49.9, I50.0, I50.11, I50.12, I50.13, I50.14, I50.19, I50.9, I51.0, I51.1, I51.2, I51.3, I51.4, I51.5, I51.6, I51.7, I51.8, I51.9, I52.0, I52.1, I52.8, Q20.0, Q20.1, Q20.2, Q20.3, Q20.4, Q20.5, Q20.6, Q20.8, Q20.9, Q21.0, Q21.1, Q21.2, Q21.3, Q21.4, Q21.8, Q21.9, Q22.0, Q22.1, Q22.2, Q22.3, Q22.4, Q22.5, Q22.6, Q22.8, Q22.9, Q23.0, Q23.1, Q23.2, Q23.3, Q23.4, Q23.8, Q23.9, Q24.0, Q24.1, Q24.2, Q24.3, Q24.4, Q24.5, Q24.6, Q24.8, Q24.9, Q25.0, Q25.1, Q25.2, Q25.3, Q25.4, Q25.5, Q25.6, Q25.7, Q25.8, Q25.9
Atherosclerosis	I69.8, I70.0, I70.1, I70.2, I70.8, I70.9
Pulmonary disease	J43.1, J43.2, J43.8, J43.9, J44.00, J44.01, J44.02, J44.03, J44.09, J44.10, J44.11, J44.12, J44.13, J44.19, J44.80, J44.81, J44.82, J44.83, J44.89, J44.90, J44.91, J44.92,

	J44.93, J44.99, J45.0, J45.1, J45.8, J45.9
Stomach and duodenal ulcers and inflammation	K25.0, K25.1, K25.2, K25.3, K25.4, K25.5, K25.6, K25.7, K25.9, K26.0, K26.1, K26.2, K26.3, K26.4, K26.5, K26.6, K26.7, K26.9, K27.0, K27.1, K27.2, K27.3, K27.4, K27.5, K27.6, K27.7, K27.9, K28.0, K28.1, K28.2, K28.3, K28.4, K28.5, K28.6, K28.7, K28.9, K29.0, K29.1, K29.2, K29.3, K29.4, K29.5, K29.6, K29.7, K29.8, K29.9, K30
Intestinal diseases	K50.0, K50.1, K50.8, K50.9, K51.0, K51.2, K51.3, K51.4, K51.5, K51.8, K51.9, K52.0, K52.1, K52.2, K52.3, K52.8, K52.9, K55.0, K55.1, K55.2, K55.3, K55.8, K55.9, K90.0
Liver diseases	K70.0, K70.1, K70.2, K70.3, K70.4, K70.9, K71.0, K71.1, K71.2, K71.3, K71.4, K715, K71.6, K71.7, K71.8, K71.9, K72.0, K72.1, K72.9, K73.0, K73.1, K73.2, K73.8, K73.9, K74.0, K74.1, K74.2, K74.3, K74.4, K74.5, K74.6, K75.0, K75.1, K75.2, K75.3, K75.4, K75.8, K75.9, K76.0, K76.1, K76.2, K76.3, K76.4, K76.5, K76.6, K76.7, K76.8, K76.9, K77.0, K77.8
Kidney disease	N00,0, N00.1, N00.2, N00.3, N00.4, N00.5, N00.6, N00.7, N00.8, N00.9, N01.0, N01.1, N01.2, N01.3, N01.4, N01.5, N01.6, N01.7, N01.8, N01.9, N02.0, N02.1, N02.2, N02.3, N02.4, N02.5, N02.6, N02.7, N02.8, N02.9, N03.0, N03.1, N03.2, N03.3, N03.4, N03.5, N03.6, N03.7, N03.8, N03.9, N04.0, N04.1, N04.2, N04.3, N04.4, N04.5, N04.6, N04.7, N04.8, N04.9, N05.0, N05.1, N05.2, N05.3, N05.4, N05.5, N05.6, N05.7, N05.8, N05.9, N06.0, N06.1, N06.2, N06.3, N06.4, N06.5, N06.6, N06.7, N06.8, N06.9, N07.0, N07.1, N07.2, N07.3, N07.4, N07.5, N07.6, N07.7, N07.8, N07.9, N08.0, N08.1, N08.2, N08.3, N08.4, N08.5, N08.8, N10, N11.0, N11.1, N11.8, N11.9, N12, N13.0, N13.1, N13.2, N13.3, N13.4, N13.5, N13.6, N13.7, N13.8, N13.9, N14.0, N14.1, N14.2, N14.3, N14.4, N15.0, N15.1, N15.8, N15.9, N16.0, N16.1, N16.2, N16.3, N16.4, N16.5, N16.8, N17.0, N17.1, N17.2, N17.8, N17.9, N18.1, N18.2, N18.3, N18.4, N18.5, N18.9, N19, N20.0
Ischemic cardiomyopathies (CMP)	I20.0, I20.1, I20.8, I20.9, I21.0, I21.1, I21.2, I21.3, I21.4, I21.9, I22.0, I22.1, I22.8, I22.9, I23.0, I23.1, I23.2, I23.3, I23.4, I23.5, I23.6, I23.8, I24.0, I24.1, I24.8, I24.9, I25.0, I25.1, I25.2, I25.3, I25.4, I25.5, I25.6, I25.8, I25.9
Malignant diseases	C00.0, C00.1, C00.2, C00.3, C00.4, C00.5, C00.6, C00.8, C00.9, C01, C02.0, C02.1, C02.2, C02.3, C02.4, C02.8, C02.9, C03.0, C03.1, C03.9, C04.0, C04.1, C04.8, C04.9, C05.0, C05.1, C05.2, C05.8, C05.9, C06.0, C06.1, C06.2, C06.8, C06.9, C07 , C08.0, C08.1, C08.8, C08.9, C09.0, C09.1, C09.8, C09.9, C10.0, C10.1, C102., C10.3, C10.4, C10.8, C10.9, C11.0, C11.1, C11.2, C11.3, C11.8, C11.9, C12, C13.0, C13.1, C13.2, C13.8, C13.9, C14.0, C14.2, C14.8, C15.0, C15.1, C15.2, C15.3, C15.4, C15.5, C15.8, C15.9, C16.0, C16.1, C16.2, C16.3, C16.4, C16.5, C16.6, C16.8, C16.9, C17.0, C17.1, C17.2, C17.3, C17.8, C17.9, C18.0, C180.1, C18.02, C180.3, C18.04, C18.1, C18.11, C18.12, C18.13, C18.14, C182., C18.21, C18.22, C18.23, C18.24, C18.3, C18.31, C18.32, C18.33, C18.34, C18.4, C18.41, C18.42, C18.43, C18.44, C18.5, C18.51, C18.52, C18.53, C18.54, C18.6, C18.61, C18.62, C18.63, C18.64, C18.7, C18.71, C18.72, C18.73, C18.74, C18.8, C18.81, C18.82, C18.83, C18.84, C18.9, C18.91, C18.92, C18.93, C18.94, C19, C19.1, C19.2, C19.3, C19.4, C20, C20.1, C20.2, C20.3, C20.4, C21.0, C21.1, C21.2, C21.8, C22.0, C22.1, C22.2, C22.3, C22.4, C22.7, C22.9, C23, C24.0, C24.1, C24.8, C24.9, C25.0, C25.1, C25.2, C25.3, C25.4, C25.7, C25.8, C25.9, C26.0, C26.1, C26.8, C26.9, C30.0, C30.1, C31.0, C31.1, C31.2, C31.3, C31.8, C31.9, C32.0, C32.1, C32.2, C32.3, C32.8, C32.9, C33, C34.0, C34.1, C34.2, C34.3, C34.8, C34.9, C37, C38.0, C38.1, C38.2, C38.3, C38.4, C38.8, C39.0, C39.8, C39.9, C40.0, C40.1, C40.2, C40.3, C40.8, C40.9, C41.0, C41.1, C41.2, C41.3, C41.4, C41.8, C41.9, C43.0, C43.1, C43.2, C43.3, C43.4, C43.5, C43.6, C43.7, C43.8, C43.9, C44.0, C44.1, C44.2, C44.3, C44.4, C44.5, C44.6, C44.7, C44.8, C44.9, C45.0, C45.1, C45.2, C45.7, C45.9, C46.0, C46.1, C46.2, C46.3, C46.7, C46.8, C46.9, C47.0, C47.1, C47.2, C47.3, C47.4, C47.5, C47.6, C47.8, C47.9, C48.0, C48.1, C48.2, C48.8, C49.0, C49.1, C49.2, C49.3, C49.4, C49.5, C49.6, C49.8, C49.9, C50.0, C50.1, C50.2, C50.3, C50.4, C50.5, C50.6, C50.8, C50.9, C51.0, C51.1, C51.2, C51.8, C51.9, C52, C53.0, C53.1, C53.8, C53.9, C54.0, C54.1, C54.2, C54.3, C54.8, C54.9, C55, C56, C57.0, C57.1, C57.2, C57.3, C57.4, C57.7, C57.8, C57.9, C58, C60.0, C60.1, C60.2, C60.8, C609., C61, C62.0, C62.1, C62.9, C63.0, C63.1, C63.2, C63.7, C63.8, C63.9, C64, C65, C66, C67.0, C67.1, C67.2, C67.3, C67.4, C67.5, C67.6, C67.7, C67.8, C67.9, C68.0, C68.1, C68.8, C68.9, C69.0, C69.1, C69.2, C69.3, C69.4, C69.5, C69.6, C69.8, C69.9, C70.0, C70.1, C70.9, C71.0, C71.1, C71.2, C71.3, C71.4, C71.5, C71.6, C71.7, C71.8, C71.9, C72.0, C72.1, C72.2, C72.3, C72.4, C72.5, C72.8, C72.9, C73, C740., C74.1, C749., C75.0, C75.1, C75.2,

	C75.3, C75.4, C75.5, C75.8, C75.9, C76.0, C76.1, C76.2, C76.3, C76.4, C76.5, C76.7, C76.8, C77.0, C77.1, C77.2, C77.3, C77.4, C77.5, C77.8, C77.9, C78.0, C78.1, C78.2, C78.3, C78.4, C78.5, C78.6, C78.7, C78.8, C79.0, C79.1, C79.2, C79.3, C79.4, C79.5, C79.6, C79.7, C79.8, C79.9, C80.0, C80.9, C81.0, C81.1, C81.2, C81.3, C81.4, C81.7, C81.9, C82.0, C82.1, C82.2, C82.3, C82.4, C82.5, C82.6, C82.7, C82.9, C83.0, C83.1, C83.3, C83.5, C83.7, C83.8, C83.9, C84.0, C84.1, C84.4, C845., C84.6, C84.7, C84.8, C84.9, C85.1, C85.2, C85.7, C85.9, C86.0, C86.1, C86.2, C86.3, C86.4, C86.5, C86.6, C88.0, C88.2, C88.3, C88.4, C887., C88.9, C90.0, C90.1, C90.2, C90.3, C91.0, C91.1, C91.3, C91.4, C91.5, C91.6, C91.7, C91.8, C91.9, C92.0, C92.1, C92.2, C92.3, C92.4, C92.5, C92.6, C92.7, C92.8, C92.9, C93.0, C93.1, C93.3, C93.7, C93.9, C94.0, C94.2, C94.3, C94.4, C94.6, C94.7, C95.0, C95.1, C95.7, C95.9, C96.0, C96.2, C96.4, C96.5, C96.6, C96.7, C96.8, C96.9, C97

Table S6: ICD-10 codes for comorbidities

The binary confounder "combined surgery" was defined as 1 if one of the MEL-codes in Table S7 was documented additionally to index surgery (SMMVR, SBMVR, MVRe) at date of index surgery.

MEL Code	Description Combined Surgery
DB050	Reconstruction of the tricuspid valvev
DB120	Replacement of the tricuspid valve with a stented valve
DB122	Replacement of the tricuspid valve with an artificial mechanical valve
DE040	Catheter examination of the cardiac conduction system
DE050	Catheter ablation of the cardiac conduction system
DE060	Catheter ablation of the cardiac conduction system in the area of the pulmonary veins
DE062	Surgical ablation of the cardiac conduction system in the area of the pulmonary veins
DE080	Implantation of a pacemaker, single-chamber system
DE081	Implantation of a pacemaker, single-chamber system, MRI-compatible
DE090	Implantation of a pacemaker, dual-chamber system
DE100	Implantation of a system for cardiac resynchronization therapy
DG060	Implantation of an intra-aortic balloon pump
DL030	Short-term circulatory support with centrifugal pump or axial flow pump
GL050	ECMO – extracorporeal membrane oxygenation
GL060	Implantation of a system for extracorporeal membrane oxygenation
XN110	Implantation of a permanent embolic protection system in the left atrial appendage
DD120	single coronary artery bypass without cardiopulmonary bypass
DD130	single coronary artery bypass with cardiopulmonary bypass
DD140	multiple coronary artery bypass without cardiopulmonary bypass
DD150	multiple coronary artery bypass with cardiopulmonary bypass
DD160	multiple coronary artery bypass with cardiopulmonary bypass
DD170	multiple coronary artery bypass with arterial free graft with CPB
DD180	single coronary artery bypass without cardiopulmonary bypass – minimally invasive
DD190	multiple coronary artery bypass with cardiopulmonary bypass – minimally invasive

Table S7: MEL-Codes for definition of confounder "combined surgery"

4.) **Statistical Analyses**

The goal of the study was to evaluate the association between the heart valve type (SMMVR, SBMVR and MVRe, see Table S4) and the following endpoints (detailed definitions see Table S1 – S3):

Primary Outcome:
- Time until all-cause death

Secondary Outcomes:
- Time until the combined endpoint major adverse cardiac and cerebrovascular event (MACCE)
- Time until the combined endpoint death or reoperation
- Time until reoperation
- Time until heart failure
- Time until myocardial infarction
- Time until embolic stroke or ICH
- Time until bleeding other than embolic stroke or ICH

All p-values smaller than 0.05 were considered as statistically significant. Due to the retrospective and exploratory character of the study no correction for multiplicity was applied for the secondary outcomes. Results on secondary outcomes may therefore be handled with care.

Categorical variables are shown as counts and percentages, while continuous variables are summarized using medians as well as 1st and 3rd quartiles. Standardized mean differences were calculated for all group comparisons to evaluate imbalance in comorbidities between groups (SMMVR, SBMVR, MVRe).

4.1. **Analyses of primary outcome**

The association between surgery type (SMMVR, SBMVR, MVRe) and the primary outcome, time until death, was first evaluated using a multivariable cox proportional hazards model. This model was accounting for surgery type groups, age, sex, combination-surgery (yes/no) and the following diagnosis groups: Infectious diseases, Diabetes mellitus, Adiposity, Hyperlipidemia, Hyperuricemia/gout (ref: no), Valvular, rhythmological, and other cardiomyopathies (CMPs), Atherosclerosis, Pulmonary disease, Stomach and duodenal ulcers and inflammation, Intestinal diseases, Liver diseases, Kidney disease, Ischemic cardiomyopathies (CMP) and Malignant diseases. To evaluate possible different trends over time, the cox model was calculated accounting for time dependent coefficients of surgery type for the time intervals 0-2, 2-4 and >4 years. Furthermore, to account for a potential imbalance between the three groups, inverse probability of treatment weighting (IPTW) was performed. Weighting was based on the inverse of a propensity score. The propensity score was calculated using a multinomial logistic regression model including the following co-variables: age, sex and above listed diagnosis groups. The cox model was calculated using corresponding weights from the propensity score. Weighted Kaplan-Meier curves were drawn using the Aalen-Johanson estimator. The model was repeated additionally accounting for the interaction between surgery type and age to evaluate varying trends between surgery types with increasing age. To illustrate the results in more detail, weighted and unweighted Kaplan-Meier curves were plotted and the number of patients at risk, the number of censored patients and the number of events is presented for each year up to the 10-year follow up. Furthermore, unweighted survival probabilities (and corresponding 95% confidence intervals) were estimated using the Kaplan-Meier method.
Proportional hazard assumption was evaluated using Schönfeld residuals, collinearity was evaluated using variance inflation factors. Results of the multivariable Cox proportional hazard model are presented as hazard ratios and corresponding 95% confidence intervals as well as p-values. HR are presented as SBMVR vs. SMMVR (reference), SBMVR vs. MVRe (reference), and SMMVR vs. MVRe (reference) thus a HR smaller than 1 indicates an increased probability of the corresponding event in the reference group. Follow-up times were calculated using the reverse Kaplan-Meier survival curve.

4.2. **Analyses of secondary outcomes**

The combined secondary outcomes MACCE as well as "death or reoperation" were analyzed similar to the primary endpoint “all-cause death”.

Time to reoperation, myocardial infarction, heart failure, stroke or ICH and bleeding other than ICH were investigated between groups using cause-specific proportional hazard regression accounting for surgery type groups, age, sex, combination-surgery (yes/no) and the diagnosis groups as for the primary parameter. Similar to the primary analysis, the cause-specific proportional hazard model was calculated accounting for time dependent

coefficients of surgery type for the time intervals 0-2, 2-4 and >4 years and the model was calculated using inverse probability of treatment weighting. The model was repeated additionally accounting for the interaction between surgery type and age. Weighted cumulative incidence curves were drawn using the Aalen-Johanson estimator. In addition, unweighted cumulative incidence curves are shown. Further note, that in the analyses for "heart failure", patients with a heart failure before Index-OP were excluded from the analyses to evaluate "newly diagnosed heart failures".

4.3. **Software**

All analyses were performed using R, version 5.1.4., using R-packages survival (version 3.8.3), survminer (version 0.5.0), prodlim (2025.4.28) and WeightIT (1.4.0) for time-to event models and inverse probability weighting.

5.) Pre-existing medicaments

	All data			SMDs		
Variables	SMMVR (n=413)	SBMVR (n=487)	MVRe (n=2620)	SMMVR vs. SBMVR	SMMVR vs. MVRe	SBMVR vs. MVRe
**Medication for hepatobiliary diseases**	3 (0.73%)	2 (0.41%)	8 (0.31%)	-0.042	-0.059	-0.018
**Intestinal anti-infectives**	3 (0.73%)	10 (2.05%)	39 (1.49%)	0.114	0.073	-0.043
**Intestinal antiphlogistics**	1 (0.24%)	1 (0.21%)	3 (0.11%)	-0.008	-0.030	-0.023
**Insulin**	13 (3.15%)	20 (4.11%)	37 (1.41%)	0.051	-0.116	-0.165
**Non-insulin antidiabetics**	42 (10.17%)	69 (14.17%)	146 (5.57%)	0.123	-0.171	-0.291
**Vitamin D**	20 (4.84%)	36 (7.39%)	67 (2.56%)	0.107	-0.121	-0.224
**Electrolytes**	33 (7.99%	85 (17.45%)	193 (7.37%)	0.287	-0.023	-0.310
**Vitamin K antagonists**	101 (24.46%)	99 (20.33%)	258 (9.85%)	-0.099	-0.395	-0.296
**Heparins**	143 (34.62%)	155 (31.83%)	536 (20.46%)	-0.059	-0.321	-0.261
**Inhibitors of platelet aggregation**	39 (9.44%)	81 (16.63%)	235 (8.97%)	0.215	-0.016	-0.231
**Direct oral anticoagulants**	35 (8.47%)	38 (7.8%)	303 (11.56%)	-0.025	0.103	0.127
**Iron therapy**	19 (4.6%)	28 (5.75%)	45 (1.72%)	0.052	-0.165	-0.214
**Antianemic preparations**	4 (0.97%)	8 (1.64%)	15 (0.57%)	0.059	-0.045	-0.102
**Glycosides**	20 (4.84%)	32 (6.57%)	58 (2.21%)	0.075	-0.143	-0.214
**Anti-arrhythmic agents**	42 (10.17%)	30 (6.16%)	142 (5.42%)	-0.147	-0.178	-0.032
**Adrenergic and dopaminergic stimulants**	2 (0.48%)	5 (1.03%)	16 (0.61%)	0.063	0.017	-0.046
**Vasodilators**	21 (5.08%)	46 (9.45%)	151 (5.76%)	0.169	0.030	-0.139
**Antihypertensive drugs**	80 (19.37%)	126 (25.87%)	420 (16.03%)	0.156	-0.088	-0.244
**New pulmonary hypertension therapy**	0 (0%)	1 (0.21%)	1 (0.04%)	0.064	0.028	-0.048
**Loop diuretics**	52 (12.59%)	78 (16.02%)	195 (7.44%)	0.098	-0.172	-0.269
**Aldosterone antagonist**	56 (13.56%)	73 (14.99%)	289 (11.03%)	0.041	-0.077	-0.118
**Beta blockers**	141 (34.14%)	182 (37.37%)	769 (29.35%)	0.067	0.067	0.171
**RAAS inhibitors**	134 (32.45%)	208 (42.71%)	959 (36.6%)	0.213	0.088	-0.125
**Lipid-lowering agents**	122 (29.54%)	183 (37.58%)	742 (28.32%)	0.171	-0.027	-0.198
**Topical antibiotics**	12 (2.91%)	16 (3.29%)	45 (1.72%)	0.022	-0.079	-0.101
**Dermal steroids**	29 (7.02%)	30 (6.16%)	127 (4.85%)	-0.035	-0.092	-0.058
**Parathyroid antagonists**	2 (0.48%)	5 (1.03%)	6 (0.23%)	0.063	-0.043	-0.101
**Systemic antibiotics**	217 (52.54%)	234 (48.05%)	1202 (45.88%)	-0.090	-0.134	-0.044
**Systemic antiviral drugs**	11 (2.66%)	10 (2.05%)	56 (2.14%)	-0.040	-0.034	0.006
**Chemo-/Immunotherapies**	4 (0.97%)	7 (1.44%)	14 (0.53%)	0.043	-0.050	-0.091
**Immunosuppressives**	12 (2.91%)	18 (3.7%)	37 (1.41%)	0.044	-0.103	-0.145
**NSAIDs**	116 (28.09%)	169 (34.7%)	694 (26.49%)	0.143	-0.036	-0.179
**Gout drugs**	18 (4.36%)	31 (6.37%)	87 (3.32%)	0.089	-0.054	-0.142
**Bisphosphonates**	407 (98.55%)	465 (95.48%)	2561 (97.75%)	0.181	0.059	-0.126
**Analgetics**	71 (17.19%)	101 (20.74%)	245 (9.35%)	0.091	-0.233	-0.323
**Inhaled drugs for obstructive pulmonary diseases**	99 (23.97%)	123 (25.26%)	482 (18.4%)	0.030	-0.137	-0.167
**Xanthines and leukotriene antagonists**	4 (0.97%)	8 (1.64%)	37 (1.41%)	0.059	0.041	-0.019

Table S8: Pre-existing medication in the year before index surgery: The numbers represent the number of patients who had used a medication and the corresponding percentage of the patients in each group. RAAS, renin-angiotensin-aldosterone system; NSAIDs, non-steroidal anti-inflammatory drugs. SMDs: Standardized Mean Differences

6.) **Primary Outcome: All-Cause Death**

Figure S2: Unweighted (A) and inverse probability of treatment weighted (B) Kaplan-Meier curves for all-cause death separately for the 3 surgery types (SMMVR, SBMVR, MVRe).

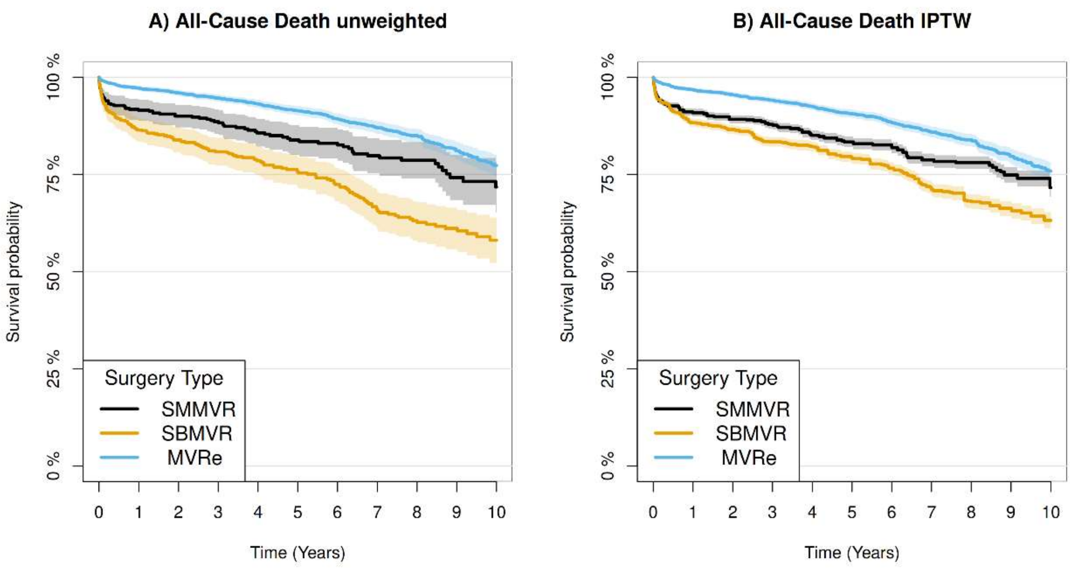

Group	Time (years)	At risk	Censored	Events	Unweighted estimate (95%-CI)	Weighted estimate (95%-CI)
**SMMVR**	**0**	413	0	0	100.00 (100.00-100.00)	100.00 (100.00-100.00)
	**1**	378	0	35	91.53 (88.84-94.21)	90.89 (89.93-91.85)
	**2**	343	29	6	90.01 (87.11-92.92)	89.18 (88.13-90.22)
	**3**	297	41	5	88.59 (85.48-91.70)	87.89 (86.78-89.00)
	**4**	248	40	9	85.70 (82.16-89.24)	85.07 (83.82-86.32)
	**5**	214	29	5	83.86 (80.04-87.67)	83.37 (82.03-84.70)
	**6**	185	26	3	82.59 (78.57-86.61)	81.91 (80.49-83.33)
	**7**	150	29	6	79.80 (75.34-84.26)	78.75 (77.16-80.33)
	**8**	115	33	2	78.64 (73.97-83.32)	78.06 (76.43-79.69)
	**9**	77	33	5	74.22 (68.41-80.02)	74.87 (72.91-76.83)
	**10**	51	24	2	71.79 (65.25-78.33)	71.61 (69.26-73.96)
**SBMVR**	**0**	487	0	1	99.79 (99.39-100.00)	99.76 (99.60-99.92)
	**1**	421	0	65	86.45 (83.41-89.49)	88.31 (87.26-89.37)
	**2**	380	29	12	83.90 (80.63-87.18)	86.56 (85.44-87.68)
	**3**	328	39	13	80.85 (77.30-84.40)	83.40 (82.17-84.64)
	**4**	294	25	9	78.53 (74.77-82.29)	82.15 (80.87-83.44)
	**5**	250	34	10	75.69 (71.68-79.71)	79.26 (77.87-80.65)
	**6**	214	26	10	72.44 (68.11-76.76)	76.61 (75.11-78.10)
	**7**	175	21	18	65.96 (61.09-70.82)	71.35 (69.66-73.04)
	**8**	139	29	7	63.11 (58.01-68.20)	68.19 (66.37-70.00)
	**9**	99	36	4	61.11 (55.82-66.41)	66.28 (64.38-68.18)
	**10**	56	39	4	58.09 (52.27-63.91)	63.18 (61.08-65.29)
**MVRe**	**0**	2620	0	1	99.96 (99.89-100.00)	99.96 (99.90-100.00)
	**1**	2547	0	72	97.21 (96.58-97.84)	96.81 (96.23-97.39)
	**2**	2310	206	31	95.98 (95.23-96.74)	95.52 (94.83-96.21)
	**3**	2001	279	30	94.66 (93.78-95.54)	94.11 (93.31-94.90)
	**4**	1706	268	29	93.19 (92.17-94.21)	92.51 (91.60-93.43)
	**5**	1392	282	30	91.42 (90.25-92.60)	90.65 (89.60-91.70)
	**6**	1137	226	29	89.31 (87.93-90.69)	88.43 (87.20-89.65)
	**7**	918	192	27	86.99 (85.38-88.59)	85.86 (84.44-87.29)
	**8**	702	197	19	85.01 (83.22-86.80)	83.93 (82.35-85.51)
	**9**	484	190	28	81.09 (78.86-83.32)	79.69 (77.73-81.65)
	**10**	275	191	18	77.31 (74.57-80.05)	75.87 (73.49-78.25)

Table S9: Number of patients at risk, number of events, number of censored patients as well as unweighted and IPT-weighted estimated survival probabilities and corresponding 95% confidence intervals, separately for years and the 3 surgery types (SMMVR, SBMVR, MVRe).

Parameter		Hazard Ratio	Lower 95%-CI	Upper 95%-CI	p-value
**Group comparisons:**	**Time interval**				
SBMVR vs. SMMVR (ref)	0-2 years	1.226	0.783	1.920	0.374
	2-4 years	1.217	0.561	2.638	0.619
	> 4 years	1.941	1.112	3.388	0.020
SBMVR vs. MVRe (ref)	0-2 years	3.211	2.272	4.540	<0.001
	2-4 years	1.774	0.986	3.193	0.056
	> 4 years	1.744	1.234	2.465	0.002
SMMVR vs. MVRe (ref)	0-2 years	2.620	1.774	3.870	<0.001
	2-4 years	1.458	0.785	2.708	0.233
	> 4 years	0.898	0.550	1.467	0.668
**Investigated confounder:**					
Age at surgery		1.017	0.999	1.036	0.067
Sex: F (ref: M)		0.897	0.697	1.154	0.399
Combined surgery (ref: no)		1.688	1.256	2.268	0.001
Infectious diseases (ref: no)		1.321	0.792	2.204	0.286
Diabetes mellitus (ref: no)		2.172	1.608	2.933	<0.001
Adiposity (ref: no)		1.078	0.699	1.663	0.733
Hyperlipidemia (ref: no)		0.566	0.409	0.783	0.001
Hyperuricemia/gout (ref: no)		0.940	0.500	1.768	0.848
Valvular, rhythmological, and other cardiomyopathies (CMPs) (ref: no)		0.712	0.519	0.976	0.035
Atherosclerosis (ref: no)		2.502	1.440	4.347	0.001
Pulmonary disease (ref: no)		0.861	0.500	1.482	0.589
Stomach and duodenal ulcers and inflammation (ref: no)		0.818	0.467	1.431	0.481
Intestinal diseases (ref: no)		1.341	0.570	3.157	0.502
Liver diseases (ref: no)		1.911	1.105	3.306	0.021
Kidney disease (ref: no)		2.450	1.711	3.508	<0.001
Ischemic cardiomyopathies (CMP) (ref: no)		0.841	0.646	1.095	0.199
Malignant diseases (ref: no)		1.091	0.507	2.347	0.825
**Interaction effects Surgery Type x Age:**					
SBMVR vs. SMMVR (ref)		0.980	0.944	1.017	0.281
SBMVR vs. MVRe (ref)		0.964	0.938	0.991	0.009
SMMVR vs. MVRe (ref)		0.984	0.952	1.017	0.343

Table S10: Hazard ratios (HRs) and corresponding 95% confidence intervals (CIs) from IPTW multivariable cox regression model accounting for all listed confounders for all-cause mortality.

Figure S3: Unweighted Kaplan-Meier curves for all-cause death separately for the 3 surgery types (SMMVR, SBMVR, MVRe) and two age sub-groups (A: <65 years; B: 65-70 years).

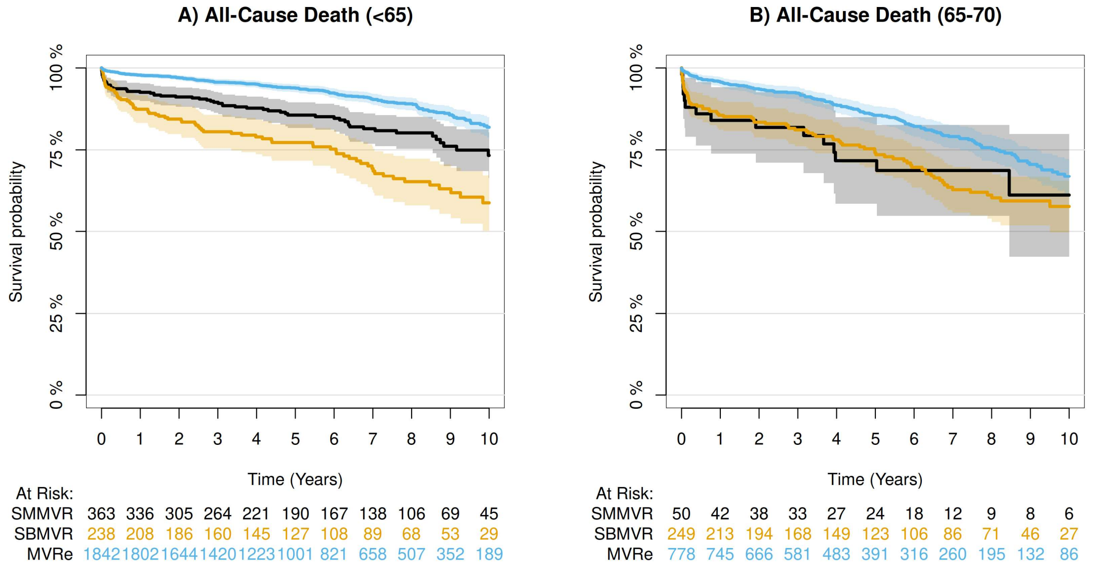

		Patients <65 years				Patiens 65-70			
**Group**	**Time (years)**	**At risk**	**Cen-sored**	**Eve nts**	**Unweighted estimate (95%-CI)**	**At risk**	**Cen-sored**	**Eve nts**	**Unweighted estimate (95%-CI)**
**SMMVR**	**0**	363	0	0	100.00 (100.00-100.00)	50	0	0	100.00 (100.00-100.00)
	**1**	336	0	27	92.56 (89.86-95.26)	42	0	8	84.00 (73.84-94.16)
	**2**	305	26	5	91.14 (88.20-94.07)	38	3	1	81.85 (71.10-92.59)
	**3**	264	36	5	89.52 (86.31-92.73)	33	5	0	81.85 (71.10-92.59)
	**4**	221	38	5	87.73 (84.22-91.23)	27	2	4	71.69 (58.44-84.93)
	**5**	190	26	5	85.60 (81.72-89.49)	24	3	0	71.69 (58.44-84.93)
	**6**	167	21	2	84.61 (80.54-88.69)	18	5	1	68.70 (54.77-82.63)
	**7**	138	23	6	81.45 (76.81-86.10)	12	6	0	68.70 (54.77-82.63)
	**8**	106	30	2	80.17 (75.28-85.07)	9	3	0	68.70 (54.77-82.63)
	**9**	69	33	4	76.10 (70.03-82.16)	8	0	1	61.07 (42.30-79.83)
	**10**	45	22	2	73.30 (66.29-80.30)	6	2	0	61.07 (42.30-79.83)
**SBMVR**	**0**	238	0	1	99.58 (98.76-100.00)	249	0	0	100.00 (100.00-100.00)
	**1**	208	0	29	87.39 (83.18-91.61)	213	0	36	85.54 (81.17-89.91)
	**2**	186	15	7	84.40 (79.78-89.02)	194	14	5	83.42 (78.78-88.06)
	**3**	160	18	8	80.53 (75.40-85.66)	168	21	5	81.10 (76.16-86.04)
	**4**	145	12	3	78.95 (73.62-84.28)	149	13	6	78.07 (72.76-83.38)
	**5**	127	15	3	77.24 (71.68-82.80)	123	19	7	74.12 (68.32-79.92)
	**6**	108	16	3	75.22 (69.36-81.08)	106	10	7	69.68 (63.36-75.99)
	**7**	89	11	8	69.30 (62.62-75.99)	86	10	10	62.69 (55.67-69.71)
	**8**	68	16	5	65.14 (57.92-72.35)	71	13	2	61.07 (53.88-68.26)
	**9**	53	13	2	62.96 (55.37-70.54)	46	23	2	59.28 (51.89-66.67)
	**10**	29	21	3	58.70 (50.20-67.20)	27	18	1	57.59 (49.70-65.48)
**MVRe**	**0**	1842	0	0	100.00 (100.00-100.00)	778	0	1	99.87 (99.62-100.00)
	**1**	1802	0	40	97.83 (97.16-98.49)	745	0	32	95.76 (94.34-97.17)
	**2**	1644	144	14	97.03 (96.25-97.81)	666	62	17	93.50 (91.75-95.24)
	**3**	1420	202	22	95.65 (94.69-96.61)	581	77	8	92.32 (90.42-94.22)
	**4**	1223	191	8	95.06 (94.03-96.10)	483	77	21	88.76 (86.39-91.12)
	**5**	1001	206	14	93.92 (92.73-95.10)	391	76	16	85.54 (82.78-88.30)
	**6**	821	165	15	92.37 (90.96-93.77)	316	61	14	82.15 (78.98-85.32)
	**7**	658	147	16	90.38 (88.70-92.06)	260	45	11	79.06 (75.52-82.60)
	**8**	507	142	9	89.05 (87.18-90.91)	195	55	10	75.68 (71.72-79.64)
	**9**	352	138	17	85.66 (83.26-88.06)	132	52	11	70.55 (65.83-75.27)
	**10**	189	151	12	81.90 (78.78-85.02)	86	40	6	66.83 (61.49-72.17)

Table S11: Number of patients at risk, number of events, number of censored patients as well as unweighted estimated survival probabilities and corresponding 95% confidence intervals, separately for years and the 3 surgery types (SMMVR, SBMVR, MVRe).

7.) **Secondary Outcomes:**

7.1 MACCE

Figure S4: Unweighted (A) and inverse probability of treatment weighted (B) Kaplan-Meier curves for MACCE separately for the 3 surgery types (SMMVR, SBMVR, MVRe).

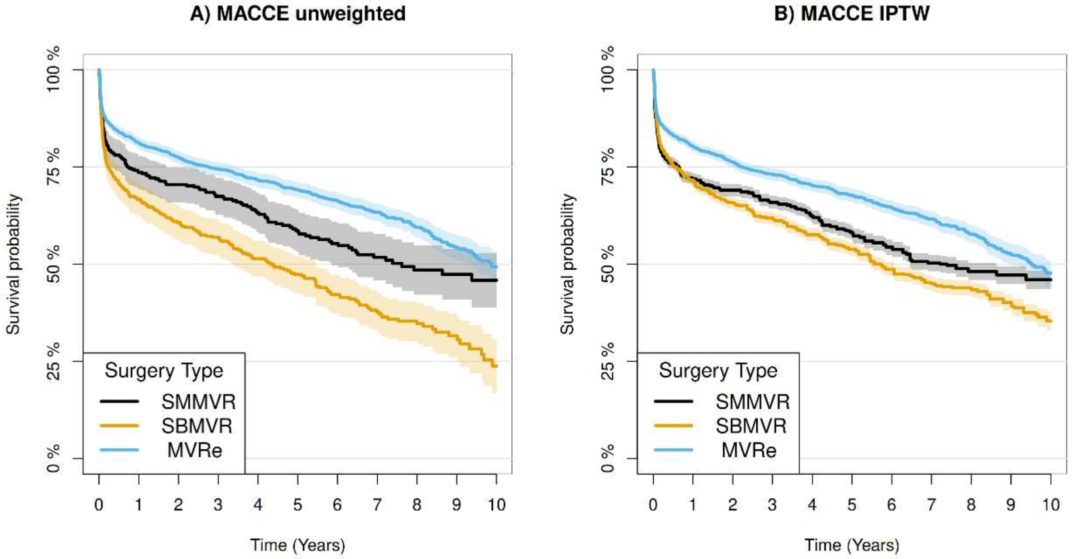

Group	Time (years)	At risk	Censored	Events	Unweighted estimate (95%-CI)	Weighted estimate (95%-CI)
**SMMVR**	**0**	413	0	0	100.00 (100.00-100.00)	100.00 (100.00-100.00)
	**1**	274	32	107	73.59 (69.28-77.89)	71.88 (70.36-73.41)
	**2**	227	36	11	70.46 (65.96-74.96)	69.02 (67.44-70.61)
	**3**	194	24	9	67.43 (62.71-72.15)	65.91 (64.25-67.57)
	**4**	156	27	11	63.23 (58.19-68.27)	62.36 (60.61-64.11)
	**5**	131	14	11	58.62 (53.26-63.98)	58.22 (56.37-60.07)
	**6**	104	20	7	55.30 (49.70-60.89)	54.40 (52.45-56.35)
	**7**	73	25	6	51.76 (45.85-57.68)	50.26 (48.18-52.34)
	**8**	52	17	4	48.47 (42.10-54.83)	48.11 (45.91-50.31)
	**9**	37	14	1	47.37 (40.79-53.94)	47.23 (44.96-49.49)
	**10**	14	22	1	45.84 (38.83-52.85)	46.00 (43.61-48.40)
**SBMVR**	**0**	487	0	1	99.79 (99.39-100.00)	99.76 (99.60-99.92)
	**1**	299	28	159	66.76 (62.54-70.98)	71.63 (70.14-73.11)
	**2**	250	23	26	60.70 (56.27-65.14)	65.77 (64.20-67.35)
	**3**	214	21	15	56.95 (52.40-61.50)	61.86 (60.23-63.49)
	**4**	174	20	20	51.39 (46.67-56.10)	57.55 (55.85-59.24)
	**5**	146	15	13	47.32 (42.49-52.16)	53.85 (52.10-55.60)
	**6**	116	15	15	42.16 (37.20-47.13)	48.68 (46.85-50.50)
	**7**	85	20	11	37.72 (32.63-42.81)	45.05 (43.16-46.94)
	**8**	55	24	6	34.71 (29.47-39.94)	43.43 (41.50-45.37)
	**9**	32	19	4	31.56 (25.95-37.17)	40.14 (38.04-42.23)
	**10**	13	13	6	23.77 (16.81-30.74)	35.39 (32.94-37.84)
**MVRe**	**0**	2620	0	1	99.96 (99.89-100.00)	99.96 (99.90-100.00)
	**1**	1904	232	483	81.19 (79.68-82.70)	80.30 (78.97-81.63)
	**2**	1599	223	82	77.47 (75.83-79.12)	76.29 (74.85-77.73)
	**3**	1311	229	59	74.44 (72.69-76.19)	73.08 (71.54-74.62)
	**4**	1053	213	45	71.63 (69.76-73.50)	70.26 (68.63-71.89)
	**5**	843	177	33	69.15 (67.16-71.14)	67.70 (65.97-69.43)
	**6**	676	135	32	66.31 (64.17-68.45)	64.57 (62.71-66.44)
	**7**	485	164	27	63.35 (61.04-65.67)	61.62 (59.61-63.63)
	**8**	319	141	25	59.48 (56.85-62.12)	57.69 (55.42-59.96)
	**9**	182	114	23	54.26 (51.10-57.43)	52.53 (49.82-55.24)
	**10**	78	92	12	49.30 (45.34-53.26)	47.67 (44.30-51.04)

Table S12: Number of patients at risk, number of events, number of censored patients as well as unweighted and IPT-weighted estimated probabilities of MACCE-free survival and corresponding 95% confidence intervals, separately for years and the 3 surgery types (SMMVR, SBMVR, MVRe).

Parameter		Hazard Ratio	Lower 95%-CI	Upper 95%-CI	p-value
**Group comparisons:**	**Time interval**				
SBMVR vs. SMMVR (ref)	0-2 years	1.075	0.819	1.412	0.600
	2-4 years	1.417	0.769	2.611	0.264
	> 4 years	1.359	0.803	2.299	0.254
SBMVR vs. MVRe (ref)	0-2 years	1.577	1.295	1.920	<0.001
	2-4 years	1.756	1.120	2.753	0.014
	> 4 years	1.673	1.175	2.382	0.004
SMMVR vs. MVRe (ref)	0-2 years	1.466	1.177	1.826	0.001
	2-4 years	1.239	0.747	2.055	0.406
	> 4 years	1.231	0.785	1.930	0.365
**Investigated Confounder:**					
Age at surgery		1.024	1.012	1.037	<0.001
Sex: F (ref: M)		0.971	0.824	1.145	0.729
Combined surgery (ref: no)		1.414	1.174	1.702	<0.001
Infectious diseases (ref: no)		1.479	1.060	2.062	0.021
Diabetes mellitus (ref: no)		1.728	1.401	2.131	<0.001
Adiposity (ref: no)		1.053	0.781	1.419	0.734
Hyperlipidemia (ref: no)		0.862	0.689	1.078	0.194
Hyperuricemia/gout (ref: no)		1.150	0.741	1.786	0.533
Valvular, rhythmological, and other cardiomyopathies (CMPs) (ref: no)		0.677	0.546	0.839	<0.001
Atherosclerosis (ref: no)		1.690	1.083	2.638	0.021
Pulmonary disease (ref: no)		1.166	0.825	1.649	0.384
Stomach and duodenal ulcers and inflammation (ref: no)		0.921	0.644	1.317	0.653
Intestinal diseases (ref: no)		0.858	0.424	1.736	0.671
Liver diseases (ref: no)		1.342	0.864	2.083	0.190
Kidney disease (ref: no)		1.837	1.420	2.377	<0.001
Ischemic cardiomyopathies (CMP) (ref: no)		0.938	0.782	1.125	0.492
Malignant diseases (ref: no)		1.300	0.684	2.468	0.423
**Interaction effects Surgery Type x Age:**					
SBMVR vs. SMMVR (ref)		1.003	0.976	1.030	0.842
SBMVR vs. MVRe (ref)		0.995	0.974	1.015	0.602
SMMVR vs. MVRe (ref)		0.992	0.971	1.013	0.454

Table S13: Hazard ratios (HRs) and corresponding 95% confidence intervals (CIs) from IPTW multivariable cox regression model accounting for all listed confounders for MACCE.

7.2 Re-operation

Figure S5: Unweighted (A) and inverse probability of treatment weighted (B) cumulative incidence curves for reoperation separately for the 3 surgery types (SMMVR, SBMVR, MVRe).

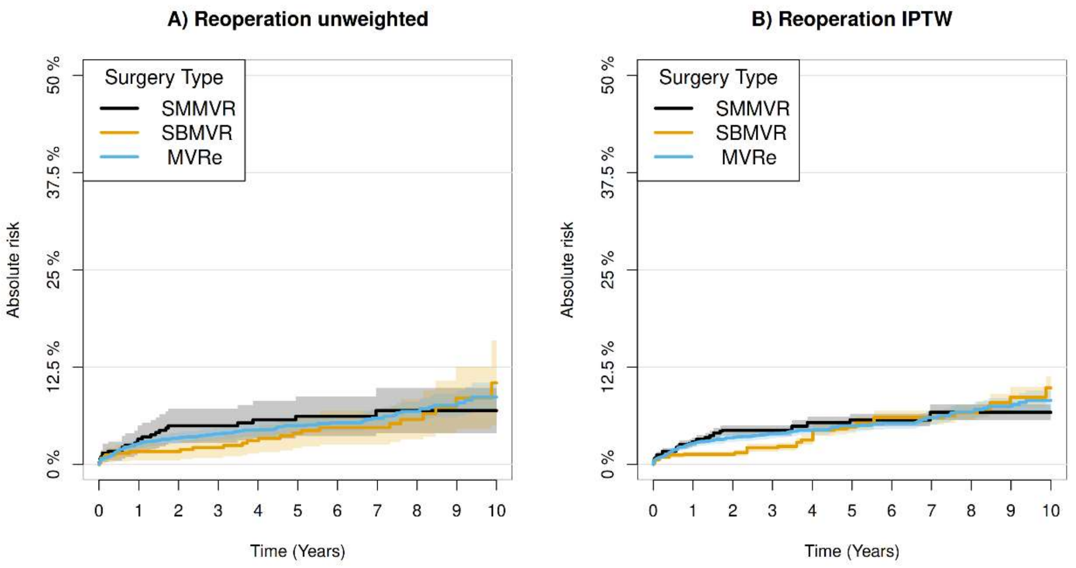

Group	Time (years)	At risk	Censored	Events	Unweighted estimate (95%-CI)	Weighted estimate (95%-CI)
**SMMVR**	**0**	413	0	0	0.00 (0.00-0.00)	0.00 (0.00-0.00)
	**1**	333	33	13	3.27 (1.52-5.02)	2.90 (2.35-3.45)
	**2**	278	44	6	4.96 (2.78-7.15)	4.38 (3.70-5.06)
	**3**	240	35	0	4.96 (2.78-7.15)	4.38 (3.70-5.06)
	**4**	199	32	2	5.74 (3.32-8.15)	5.38 (4.59-6.16)
	**5**	171	22	1	6.19 (3.63-8.75)	5.71 (4.89-6.54)
	**6**	138	30	0	6.19 (3.63-8.75)	5.71 (4.89-6.54)
	**7**	99	32	1	6.91 (4.01-9.82)	6.70 (5.71-7.69)
	**8**	71	28	0	6.91 (4.01-9.82)	6.70 (5.71-7.69)
	**9**	48	20	0	6.91 (4.01-9.82)	6.70 (5.71-7.69)
	**10**	22	26	0	6.91 (4.01-9.82)	6.70 (5.71-7.69)
**SBMVR**	**0**	487	0	0	0.00 (0.00-0.00)	0.00 (0.00-0.00)
	**1**	378	41	8	1.66 (0.52-2.81)	1.29 (0.94-1.65)
	**2**	334	32	0	1.66 (0.52-2.81)	1.29 (0.94-1.65)
	**3**	288	33	2	2.17 (0.83-3.50)	2.14 (1.67-2.62)
	**4**	251	27	3	3.04 (1.39-4.69)	3.15 (2.55-3.74)
	**5**	214	25	3	4.02 (2.05-5.99)	4.80 (4.04-5.57)
	**6**	181	22	2	4.73 (2.54-6.91)	6.03 (5.13-6.92)
	**7**	135	31	0	4.73 (2.54-6.91)	6.03 (5.13-6.92)
	**8**	88	41	2	5.78 (3.18-8.37)	6.66 (5.70-7.62)
	**9**	48	36	3	8.54 (4.53-12.55)	8.63 (7.35-9.92)
	**10**	21	25	1	10.49 (5.07-15.91)	9.84 (8.32-11.37)
**MVRe**	**0**	2620	0	0	0.00 (0.00-0.00)	0.00 (0.00-0.00)
	**1**	2223	261	68	2.69 (2.06-3.32)	2.71 (2.23-3.19)
	**2**	1922	260	15	3.36 (2.65-4.08)	3.40 (2.86-3.94)
	**3**	1620	268	11	3.93 (3.15-4.72)	3.94 (3.35-4.53)
	**4**	1334	254	8	4.41 (3.56-5.26)	4.38 (3.74-5.01)
	**5**	1071	230	8	4.99 (4.06-5.93)	4.95 (4.25-5.64)
	**6**	867	178	4	5.35 (4.36-6.34)	5.29 (4.55-6.02)
	**7**	635	208	5	5.93 (4.82-7.04)	6.00 (5.16-6.84)
	**8**	433	187	6	6.79 (5.49-8.09)	6.76 (5.80-7.71)
	**9**	243	171	4	7.60 (6.09-9.11)	7.46 (6.39-8.54)
	**10**	107	124	3	8.65 (6.75-10.56)	8.22 (6.97-9.47)

Table S14: Number of patients at risk, number of events, number of censored patients as well as unweighted and IPT-weighted estimated probabilities of re-operation and corresponding 95% confidence intervals, separately for years and the 3 surgery types (SMMVR, SBMVR, MVRe).

Parameter		Hazard Ratio	Lower 95%-CI	Upper 95%-CI	p-value
**Group comparisons:**	**Time interval**				
SBMVR vs. SMMVR (ref)	0-2 years	0.291	0.113	0.749	0.010
	2-4 years	1.896	0.355	10.127	0.454
	> 4 years	4.685	0.945	23.229	0.059
SBMVR vs. MVRe (ref)	0-2 years	0.395	0.167	0.933	0.034
	2-4 years	1.985	0.697	5.652	0.199
	> 4 years	2.074	0.903	4.762	0.085
SMMVR vs. MVRe (ref)	0-2 years	1.355	0.787	2.336	0.273
	2-4 years	1.047	0.245	4.475	0.950
	> 4 years	0.443	0.103	1.901	0.273
**Investigated Confounder:**					
Age at surgery		1.011	0.985	1.037	0.413
Sex: F (ref: M)		0.725	0.458	1.147	0.169
Combined surgery (ref: no)		1.138	0.632	2.049	0.667
Infectious diseases (ref: no)		2.160	0.934	4.995	0.072
Diabetes mellitus (ref: no)		1.632	0.778	3.423	0.195
Adiposity (ref: no)		1.222	0.579	2.579	0.599
Hyperlipidemia (ref: no)		0.808	0.386	1.693	0.573
Hyperuricemia/gout (ref: no)		0.680	0.140	3.310	0.633
Valvular, rhythmological, and other cardiomyopathies (CMPs) (ref: no)		0.990	0.537	1.826	0.975
Atherosclerosis (ref: no)		1.071	0.289	3.967	0.918
Pulmonary disease (ref: no)		1.339	0.461	3.885	0.591
Stomach and duodenal ulcers and inflammation (ref: no)		1.097	0.436	2.760	0.845
Intestinal diseases (ref: no)					
Liver diseases (ref: no)		1.872	0.666	5.265	0.235
Kidney disease (ref: no)		0.819	0.389	1.726	0.600
Ischemic cardiomyopathies (CMP) (ref: no)		0.467	0.263	0.828	0.009
Malignant diseases (ref: no)		1.997	0.335	11.891	0.448
**Interaction effects Surgery Type x Age:**					
SBMVR vs. SMMVR (ref)		1.029	0.966	1.097	0.376
SBMVR vs. MVRe (ref)		1.031	0.977	1.089	0.261
SMMVR vs. MVRe (ref)		1.002	0.957	1.050	0.925

Table S15: Hazard ratios (HRs) and corresponding 95% confidence intervals (CIs) from IPTW multivariable cox regression model accounting for all listed confounders for reoperation.

Figure S6: Unweighted Kaplan-Meier curve for death after reoperation over all 3 surgery types.

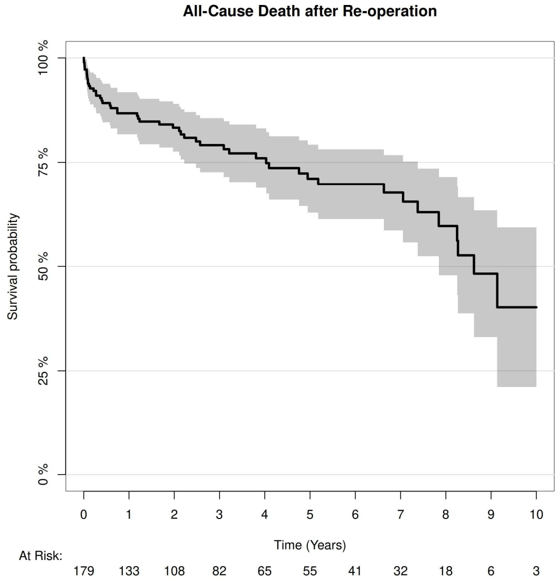

Time (years)	At risk	Censored	Events	Unweighted estimate (95%-CI)
**0**	179	0	0	0.00 (0.00-0.00)
**1**	133	23	23	13.25 (8.20-18.30)
**2**	108	20	5	16.70 (11.01-22.39)
**3**	82	21	5	20.88 (14.40-27.36)
**4**	65	14	3	24.02 (16.88-31.15)
**5**	55	6	4	28.96 (20.80-37.11)
**6**	41	13	1	30.27 (21.87-38.67)
**7**	32	8	1	32.32 (23.26-41.39)
**8**	18	11	3	40.34 (28.52-52.15)
**9**	6	9	3	51.74 (36.60-66.89)
**10**	3	2	1	59.79 (40.65-78.93)

Table S16: Number of patients at risk, number of events, number of censored patients and unweighted estimated probabilities of death after reoperation and corresponding 95% confidence intervals, separately for years.

7.3 **Myocardial Infarction**

Figure S7: Unweighted (A) and inverse probability of treatment weighted (B) cumulative incidence curves for myocardial infarction separately for the 3 surgery types (SMMVR, SBMVR, MVRe).

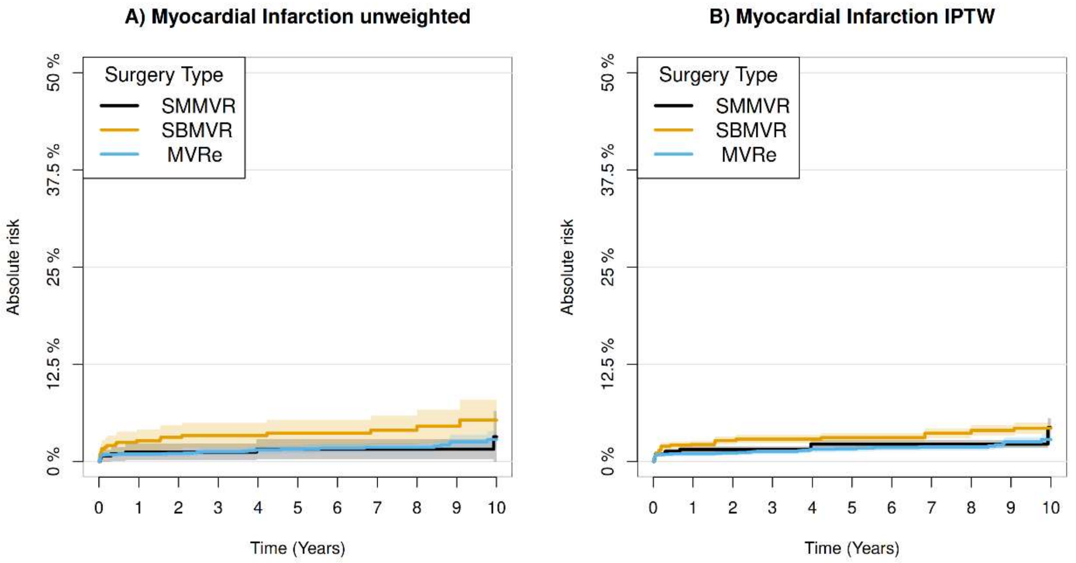

Group	Time (years)	At risk	Censored	Events	Unweighted estimate (95%-CI)	Weighted estimate (95%-CI)
**SMMVR**	**0**	413	0	0	0.00 (0.00-0.00)	0.00 (0.00-0.00)
	**1**	364	10	5	1.21 (0.16-2.27)	1.54 (1.14-1.95)
	**2**	323	35	0	1.21 (0.16-2.27)	1.54 (1.14-1.95)
	**3**	276	42	0	1.21 (0.16-2.27)	1.54 (1.14-1.95)
	**4**	229	38	1	1.58 (0.31-2.85)	2.24 (1.72-2.77)
	**5**	197	27	0	1.58 (0.31-2.85)	2.24 (1.72-2.77)
	**6**	173	21	0	1.58 (0.31-2.85)	2.24 (1.72-2.77)
	**7**	136	32	0	1.58 (0.31-2.85)	2.24 (1.72-2.77)
	**8**	97	37	0	1.58 (0.31-2.85)	2.24 (1.72-2.77)
	**9**	67	26	0	1.58 (0.31-2.85)	2.24 (1.72-2.77)
	**10**	43	21	1	3.19 (0.00-6.55)	4.34 (3.05-5.63)
**SBMVR**	**0**	487	0	0	0.00 (0.00-0.00)	0.00 (0.00-0.00)
	**1**	406	4	13	2.67 (1.24-4.10)	2.18 (1.73-2.64)
	**2**	347	45	2	3.11 (1.56-4.67)	2.69 (2.18-3.20)
	**3**	307	27	1	3.35 (1.73-4.97)	2.88 (2.35-3.41)
	**4**	269	30	0	3.35 (1.73-4.97)	2.88 (2.35-3.41)
	**5**	231	27	1	3.64 (1.93-5.35)	3.07 (2.52-3.63)
	**6**	197	24	0	3.64 (1.93-5.35)	3.07 (2.52-3.63)
	**7**	157	23	1	4.02 (2.16-5.88)	3.63 (3.00-4.27)
	**8**	118	34	1	4.53 (2.43-6.64)	4.01 (3.31-4.70)
	**9**	73	41	0	4.53 (2.43-6.64)	4.01 (3.31-4.70)
	**10**	40	30	1	5.33 (2.73-7.93)	4.28 (3.54-5.03)
**MVRe**	**0**	2620	0	0	0.00 (0.00-0.00)	0.00 (0.00-0.00)
	**1**	2455	67	25	0.95 (0.58-1.33)	1.00 (0.70-1.30)
	**2**	2146	277	2	1.04 (0.65-1.43)	1.10 (0.79-1.41)
	**3**	1853	262	5	1.27 (0.83-1.71)	1.32 (0.98-1.67)
	**4**	1546	274	4	1.50 (1.01-1.99)	1.58 (1.20-1.96)
	**5**	1260	259	1	1.57 (1.06-2.08)	1.64 (1.25-2.03)
	**6**	1035	197	2	1.72 (1.17-2.27)	1.79 (1.37-2.21)
	**7**	827	184	1	1.82 (1.23-2.40)	1.88 (1.44-2.32)
	**8**	600	210	0	1.82 (1.23-2.40)	1.88 (1.44-2.32)
	**9**	399	173	4	2.52 (1.62-3.41)	2.53 (1.90-3.16)
	**10**	207	175	1	2.83 (1.75-3.91)	2.83 (2.11-3.55)

Table S17: Number of patients at risk, number of events, number of censored patients as well as unweighted and IPT-weighted estimated probabilities of Myocardial infarction and corresponding 95% confidence intervals, separately for years and the 3 surgery types (SMMVR, SBMVR, MVRe).

Parameter		Hazard Ratio	Lower 95%-CI	Upper 95%-CI	p-value
**Group comparisons:**	**Time interval**				
SBMVR vs. SMMVR (ref)	0-2 years	1.870	0.643	5.437	0.250
	2-4 years	0.402	0.024	6.588	0.523
	> 4 years	2.399	0.372	15.460	0.357
SBMVR vs. MVRe (ref)	0-2 years	2.556	1.269	5.149	0.009
	2-4 years	0.518	0.064	4.173	0.537
	> 4 years	1.650	0.456	5.980	0.446
SMMVR vs. MVRe (ref)	0-2 years	1.367	0.537	3.477	0.512
	2-4 years	1.290	0.161	10.331	0.810
	> 4 years	0.688	0.133	3.550	0.655
**Investigated Confounder:**					
Age at surgery		1.015	0.977	1.055	0.451
Sex: F (ref: M)		0.955	0.481	1.894	0.894
Combined surgery (ref: no)		4.321	1.901	9.821	<0.001
Infectious diseases (ref: no)		2.010	0.775	5.211	0.151
Diabetes mellitus (ref: no)		2.056	0.986	4.287	0.055
Adiposity (ref: no)		0.500	0.162	1.546	0.229
Hyperlipidemia (ref: no)		0.756	0.300	1.902	0.552
Hyperuricemia/gout (ref: no)		1.185	0.309	4.548	0.805
Valvular, rhythmological, and other cardiomyopathies (CMPs) (ref: no)		0.304	0.151	0.611	0.001
Atherosclerosis (ref: no)					
Pulmonary disease (ref: no)		1.281	0.409	4.016	0.671
Stomach and duodenal ulcers and inflammation (ref: no)		1.413	0.544	3.665	0.478
Intestinal diseases (ref: no)					
Liver diseases (ref: no)		0.187	0.024	1.476	0.112
Kidney disease (ref: no)		0.913	0.421	1.978	0.817
Ischemic cardiomyopathies (CMP) (ref: no)		1.212	0.599	2.451	0.593
Malignant diseases (ref: no)					
**Interaction effects Surgery Type x Age:**					
SBMVR vs. SMMVR (ref)		0.972	0.888	1.065	0.546
SBMVR vs. MVRe (ref)		0.990	0.933	1.050	0.742
SMMVR vs. MVRe (ref)		1.018	0.937	1.107	0.669

Table S18: Hazard ratios (HRs) and corresponding 95% confidence intervals (CIs) from IPTW multivariable cox regression model accounting for all listed confounders for myocardial infarction.

7.4 Heart failure

Figure S8: Unweighted (A) and inverse probability of treatment weighted (B) cumulative incidence curves for heart failure separately for the 3 surgery types (SMMVR, SBMVR, MVRe).

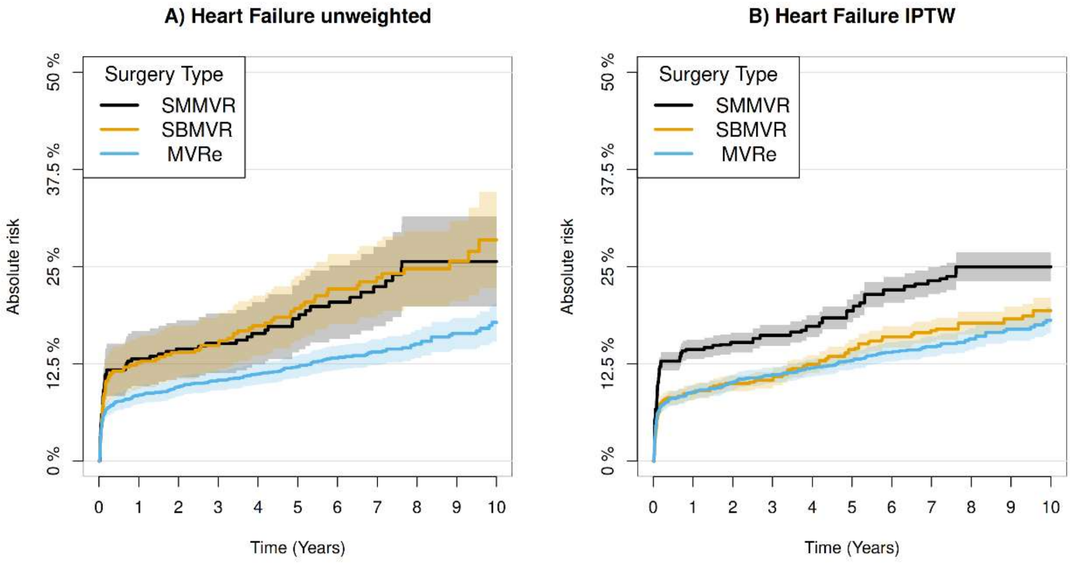

Group	Time (years)	At risk	Censored	Events	Unweighted estimate (95%-CI)	Weighted estimate (95%-CI)
**SMMVR**	**0**	350	0	0	0.00 (0.00-0.00)	0.00 (0.00-0.00)
	**1**	276	10	46	13.15 (9.61-16.69)	14.34 (13.08-15.60)
	**2**	242	28	4	14.42 (10.72-18.12)	15.25 (13.96-16.54)
	**3**	205	33	2	15.14 (11.33-18.94)	16.16 (14.83-17.49)
	**4**	170	28	3	16.41 (12.40-20.42)	17.33 (15.93-18.72)
	**5**	142	22	4	18.30 (13.97-22.63)	19.31 (17.80-20.81)
	**6**	122	16	4	20.43 (15.74-25.12)	22.00 (20.35-23.66)
	**7**	92	24	3	22.43 (17.34-27.52)	23.20 (21.47-24.93)
	**8**	63	24	4	25.66 (19.89-31.43)	24.97 (23.11-26.83)
	**9**	48	14	0	25.66 (19.89-31.43)	24.97 (23.11-26.83)
	**10**	33	14	0	25.66 (19.89-31.43)	24.97 (23.11-26.83)
**SBMVR**	**0**	389	0	0	0.00 (0.00-0.00)	0.00 (0.00-0.00)
	**1**	296	2	49	12.60 (9.30-15.90)	8.78 (7.79-9.77)
	**2**	259	25	5	13.96 (10.51-17.42)	9.98 (8.93-11.03)
	**3**	228	21	3	14.85 (11.28-18.41)	10.40 (9.33-11.47)
	**4**	196	21	8	17.41 (13.54-21.29)	12.45 (11.27-13.62)
	**5**	165	22	6	19.63 (15.47-23.78)	14.34 (13.07-15.61)
	**6**	133	21	6	22.12 (17.64-26.59)	15.97 (14.61-17.33)
	**7**	104	19	3	23.58 (18.90-28.27)	16.77 (15.36-18.18)
	**8**	76	23	2	24.72 (19.85-29.60)	17.73 (16.25-19.21)
	**9**	42	31	1	25.72 (20.54-30.89)	18.29 (16.74-19.83)
	**10**	24	15	2	28.45 (22.25-34.65)	19.34 (17.68-20.99)
**MVRe**	**0**	2252	0	0	0.00 (0.00-0.00)	0.00 (0.00-0.00)
	**1**	1975	54	191	8.49 (7.34-9.64)	8.89 (7.93-9.84)
	**2**	1703	234	21	9.52 (8.30-10.75)	10.10 (9.09-11.11)
	**3**	1457	218	16	10.41 (9.12-11.69)	11.11 (10.05-12.16)
	**4**	1208	223	12	11.19 (9.84-12.54)	11.97 (10.87-13.07)
	**5**	971	215	12	12.13 (10.69-13.56)	12.92 (11.77-14.08)
	**6**	805	148	12	13.24 (11.69-14.78)	13.97 (12.74-15.20)
	**7**	637	148	7	14.02 (12.38-15.66)	14.71 (13.42-16.00)
	**8**	456	165	7	15.05 (13.26-16.84)	15.70 (14.32-17.07)
	**9**	305	129	7	16.41 (14.38-18.44)	16.96 (15.45-18.47)
	**10**	162	133	4	17.83 (15.41-20.26)	18.10 (16.41-19.79)

Table S19: Number of patients at risk, number of events, number of censored patients as well as unweighted and IPT-weighted estimated probabilities of heart failure and corresponding 95% confidence intervals, separately for years and the 3 surgery types (SMMVR, SBMVR, MVRe).

Parameter		Hazard Ratio	Lower 95%-CI	Upper 95%-CI	p-value
**Group comparisons:**	**Time interval**				
SBMVR vs. SMMVR (ref)	0-2 years	0.617	0.406	0.937	0.023
	2-4 years	1.224	0.401	3.742	0.723
	> 4 years	0.821	0.381	1.768	0.614
SBMVR vs. MVRe (ref)	0-2 years	1.089	0.783	1.516	0.611
	2-4 years	1.581	0.738	3.388	0.238
	> 4 years	1.631	0.931	2.859	0.088
SMMVR vs. MVRe (ref)	0-2 years	1.766	1.264	2.468	0.001
	2-4 years	1.292	0.482	3.462	0.611
	> 4 years	1.987	1.033	3.822	0.040
**Investigated Confounder:**					
Age at surgery		1.042	1.028	1.057	<0.001
Sex: F (ref: M)		1.227	0.943	1.597	0.128
Combined surgery (ref: no)		1.586	1.196	2.102	0.001
Infectious diseases (ref: no)		1.912	1.142	3.200	0.014
Diabetes mellitus (ref: no)		1.798	1.240	2.608	0.002
Adiposity (ref: no)		1.495	0.928	2.406	0.098
Hyperlipidemia (ref: no)		0.933	0.661	1.317	0.694
Hyperuricemia/gout (ref: no)		1.928	0.983	3.783	0.056
Valvular, rhythmological, and other cardiomyopathies (CMPs) (ref: no)		0.527	0.384	0.724	<0.001
Atherosclerosis (ref: no)		1.686	0.672	4.231	0.266
Pulmonary disease (ref: no)		1.108	0.617	1.990	0.730
Stomach and duodenal ulcers and inflammation (ref: no)		0.949	0.503	1.791	0.872
Intestinal diseases (ref: no)		1.908	0.613	5.935	0.265
Liver diseases (ref: no)		0.584	0.236	1.449	0.246
Kidney disease (ref: no)		1.448	0.876	2.395	0.149
Ischemic cardiomyopathies (CMP) (ref: no)		1.197	0.885	1.618	0.243
Malignant diseases (ref: no)		1.301	0.503	3.370	0.587
**Interaction effects Surgery Type x Age:**					
SBMVR vs. SMMVR (ref)		1.057	1.018	1.098	0.004
SBMVR vs. MVRe (ref)		1.039	1.007	1.072	0.017
SMMVR vs. MVRe (ref)		0.983	0.952	1.014	0.269

Table S20: Hazard ratios (HRs) and corresponding 95% confidence intervals (CIs) from IPTW multivariable cox regression model accounting for all listed confounders for heart failure.

7.5 Stroke or ICH

Figure S9: Unweighted (A) and inverse probability of treatment weighted (B) cumulative incidence curves for stroke or ICH separately for the 3 surgery types (SMMVR, SBMVR, MVRe).

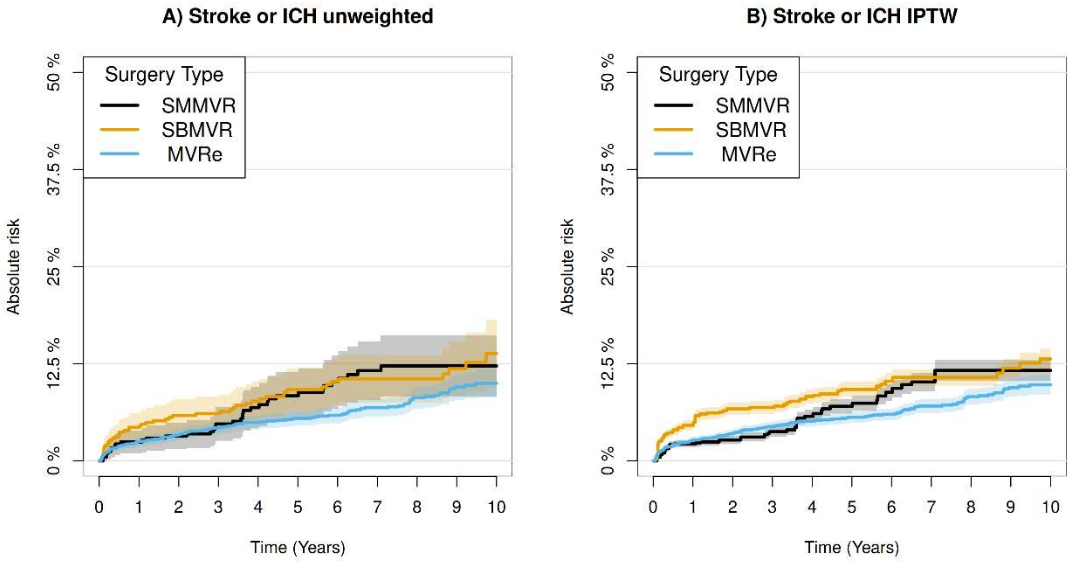

Group	Time (years)	At risk	Censored	Events	Unweighted estimate (95%-CI)	Weighted estimate (95%-CI)
**SMMVR**	**0**	413	0	0	0.00 (0.00-0.00)	0.00 (0.00-0.00)
	**1**	359	10	10	2.42 (0.94-3.90)	2.21 (1.73-2.68)
	**2**	315	35	3	3.19 (1.48-4.90)	2.68 (2.16-3.21)
	**3**	262	43	5	4.75 (2.59-6.91)	3.76 (3.12-4.40)
	**4**	212	37	6	6.86 (4.17-9.55)	5.73 (4.90-6.56)
	**5**	181	22	4	8.38 (5.35-11.41)	7.02 (6.09-7.95)
	**6**	157	18	4	10.15 (6.72-13.58)	8.82 (7.74-9.90)
	**7**	121	29	3	11.62 (7.86-15.37)	10.15 (8.97-11.34)
	**8**	86	33	1	12.22 (8.31-16.12)	11.63 (10.31-12.96)
	**9**	59	24	0	12.22 (8.31-16.12)	11.63 (10.31-12.96)
	**10**	38	19	0	12.22 (8.31-16.12)	11.63 (10.31-12.96)
**SBMVR**	**0**	487	0	0	0.00 (0.00-0.00)	0.00 (0.00-0.00)
	**1**	398	4	21	4.31 (2.51-6.12)	4.57 (3.91-5.24)
	**2**	335	44	7	5.84 (3.74-7.95)	6.70 (5.90-7.49)
	**3**	295	28	1	6.09 (3.94-8.24)	6.87 (6.07-7.68)
	**4**	254	29	6	7.69 (5.22-10.15)	8.29 (7.39-9.18)
	**5**	217	24	5	9.18 (6.43-11.93)	9.20 (8.25-10.15)
	**6**	181	24	3	10.20 (7.25-13.15)	10.26 (9.23-11.29)
	**7**	148	19	1	10.56 (7.54-13.58)	10.74 (9.67-11.80)
	**8**	114	30	0	10.56 (7.54-13.58)	10.74 (9.67-11.80)
	**9**	70	39	2	11.88 (8.39-15.36)	11.95 (10.74-13.15)
	**10**	35	30	2	13.83 (9.49-18.17)	13.12 (11.76-14.48)
**MVRe**	**0**	2620	0	0	0.00 (0.00-0.00)	0.00 (0.00-0.00)
	**1**	2419	66	65	2.49 (1.89-3.09)	2.65 (2.19-3.11)
	**2**	2095	275	21	3.37 (2.67-4.07)	3.51 (2.97-4.05)
	**3**	1799	252	19	4.25 (3.45-5.05)	4.42 (3.81-5.03)
	**4**	1495	265	13	4.95 (4.07-5.82)	5.09 (4.42-5.76)
	**5**	1210	251	8	5.47 (4.52-6.41)	5.62 (4.91-6.33)
	**6**	989	190	5	5.86 (4.86-6.86)	6.00 (5.25-6.75)
	**7**	788	171	11	6.85 (5.70-8.00)	7.06 (6.20-7.92)
	**8**	562	201	10	8.12 (6.75-9.50)	8.25 (7.25-9.25)
	**9**	367	165	8	9.51 (7.85-11.17)	9.43 (8.26-10.59)
	**10**	196	157	2	9.99 (8.21-11.77)	9.79 (8.57-11.02)

Table S21: Number of patients at risk, number of events, number of censored patients as well as unweighted and IPT-weighted estimated probabilities of stroke or ICH and corresponding 95% confidence intervals, separately for years and the 3 surgery types (SMMVR, SBMVR, MVRe).

Parameter		Hazard Ratio	Lower 95%-CI	Upper 95%-CI	p-value
**Group comparisons:**	**Time interval**				
SBMVR vs. SMMVR (ref)	0-2 years	2.547	1.142	5.680	0.022
	2-4 years	0.558	0.202	1.542	0.260
	> 4 years	0.684	0.299	1.563	0.368
SBMVR vs. MVRe (ref)	0-2 years	2.121	1.223	3.681	0.007
	2-4 years	1.126	0.487	2.604	0.782
	> 4 years	1.365	0.724	2.576	0.336
SMMVR vs. MVRe (ref)	0-2 years	0.833	0.445	1.560	0.568
	2-4 years	2.018	0.938	4.343	0.072
	> 4 years	1.997	0.990	4.030	0.054
**Investigated Confounder:**					
Age at surgery		1.016	0.986	1.047	0.296
Sex: F (ref: M)		1.214	0.829	1.778	0.320
Combined surgery (ref: no)		0.933	0.602	1.445	0.755
Infectious diseases (ref: no)		1.354	0.628	2.917	0.440
Diabetes mellitus (ref: no)		1.472	0.875	2.476	0.145
Adiposity (ref: no)		0.663	0.318	1.379	0.271
Hyperlipidemia (ref: no)		1.301	0.817	2.069	0.267
Hyperuricemia/gout (ref: no)		0.544	0.099	2.981	0.483
Valvular, rhythmological, and other cardiomyopathies (CMPs) (ref: no)		1.528	0.890	2.623	0.125
Atherosclerosis (ref: no)		0.617	0.115	3.316	0.574
Pulmonary disease (ref: no)		1.003	0.422	2.383	0.995
Stomach and duodenal ulcers and inflammation (ref: no)		0.636	0.242	1.670	0.358
Intestinal diseases (ref: no)		0.110	0.014	0.888	0.038
Liver diseases (ref: no)		1.768	0.714	4.382	0.218
Kidney disease (ref: no)		1.280	0.648	2.530	0.477
Ischemic cardiomyopathies (CMP) (ref: no)		0.798	0.519	1.227	0.305
Malignant diseases (ref: no)		0.938	0.201	4.374	0.935
**Interaction effects Surgery Type x Age:**					
SBMVR vs. SMMVR (ref)		1.012	0.948	1.081	0.724
SBMVR vs. MVRe (ref)		0.986	0.935	1.040	0.611
SMMVR vs. MVRe (ref)		0.975	0.926	1.026	0.323

Table S22: Hazard ratios (HRs) and corresponding 95% confidence intervals (CIs) from IPTW multivariable cox regression model accounting for all listed confounders for stroke or ICH.

7.6 Bleeding other than ICH

Figure S10: Unweighted (A) and inverse probability of treatment weighted (B) cumulative incidence curves for bleeding other than ICH separately for the 3 surgery types (SMMVR, SBMVR, MVRe).

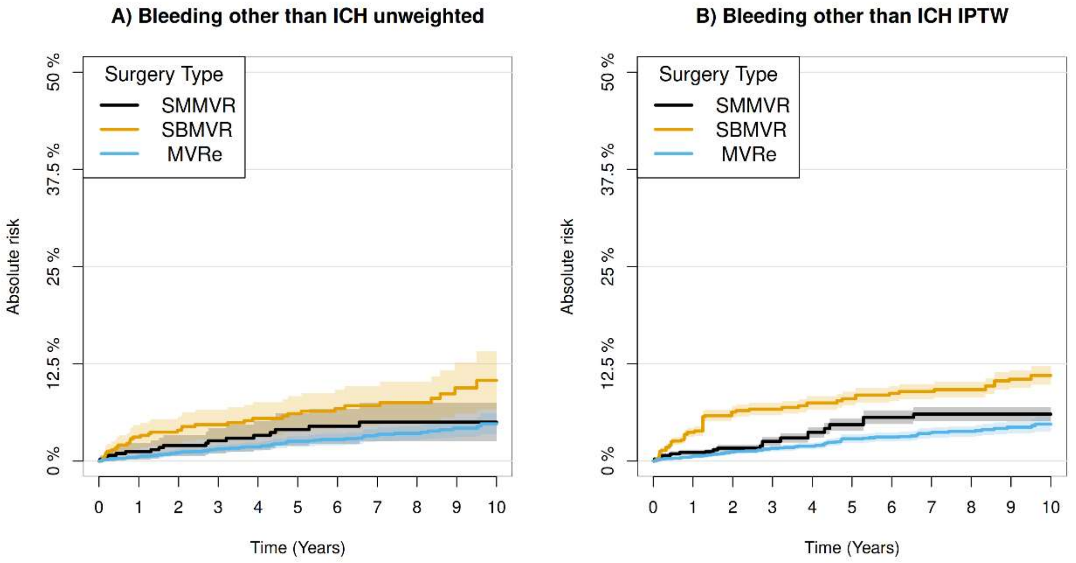

Group	Time (years)	At risk	Censored	Events	Unweighted estimate (95%-CI)	Weighted estimate (95%-CI)
**SMMVR**	**0**	413	0	0	0.00 (0.00-0.00)	0.00 (0.00-0.00)
	**1**	363	10	5	1.21 (0.16-2.27)	1.12 (0.78-1.45)
	**2**	320	35	3	1.99 (0.63-3.36)	1.65 (1.24-2.07)
	**3**	273	42	2	2.61 (1.00-4.21)	2.52 (1.99-3.05)
	**4**	228	35	2	3.30 (1.44-5.15)	3.70 (3.02-4.37)
	**5**	194	27	2	4.05 (1.94-6.17)	4.67 (3.90-5.45)
	**6**	170	20	1	4.48 (2.21-6.75)	5.62 (4.74-6.50)
	**7**	132	32	1	5.00 (2.53-7.47)	6.02 (5.09-6.95)
	**8**	94	36	0	5.00 (2.53-7.47)	6.02 (5.09-6.95)
	**9**	64	26	0	5.00 (2.53-7.47)	6.02 (5.09-6.95)
	**10**	41	21	0	5.00 (2.53-7.47)	6.02 (5.09-6.95)
**SBMVR**	**0**	487	0	0	0.00 (0.00-0.00)	0.00 (0.00-0.00)
	**1**	408	4	15	3.08 (1.55-4.62)	3.69 (3.10-4.29)
	**2**	346	47	4	3.95 (2.21-5.70)	6.30 (5.52-7.08)
	**3**	306	26	3	4.67 (2.76-6.58)	6.66 (5.86-7.47)
	**4**	266	29	3	5.48 (3.38-7.58)	7.47 (6.61-8.32)
	**5**	228	27	2	6.09 (3.84-8.34)	8.01 (7.11-8.91)
	**6**	195	24	2	6.76 (4.34-9.18)	8.69 (7.74-9.64)
	**7**	156	23	1	7.12 (4.61-9.62)	8.93 (7.97-9.90)
	**8**	122	29	1	7.52 (4.90-10.14)	9.18 (8.19-10.17)
	**9**	73	42	3	9.40 (6.07-12.73)	10.51 (9.38-11.64)
	**10**	39	31	1	10.36 (6.58-14.15)	11.00 (9.80-12.20)
**MVRe**	**0**	2620	0	0	0.00 (0.00-0.00)	0.00 (0.00-0.00)
	**1**	2467	67	15	0.58 (0.29-0.87)	0.60 (0.37-0.82)
	**2**	2145	279	12	1.08 (0.67-1.48)	1.15 (0.84-1.46)
	**3**	1846	261	10	1.56 (1.06-2.06)	1.64 (1.26-2.01)
	**4**	1539	274	5	1.83 (1.27-2.38)	1.91 (1.50-2.32)
	**5**	1244	257	11	2.54 (1.85-3.23)	2.85 (2.31-3.39)
	**6**	1023	196	3	2.77 (2.03-3.51)	3.08 (2.51-3.66)
	**7**	810	182	6	3.34 (2.48-4.21)	3.62 (2.96-4.27)
	**8**	588	206	2	3.56 (2.65-4.48)	3.81 (3.13-4.50)
	**9**	388	175	4	4.22 (3.11-5.33)	4.35 (3.55-5.16)
	**10**	204	169	2	4.77 (3.43-6.12)	4.74 (3.82-5.66)

Table S23: Number of patients at risk, number of events, number of censored patients as well as unweighted and IPT-weighted estimated probabilities of bleeding other than ICH and corresponding 95% confidence intervals, separately for years and the 3 surgery types (SMMVR, SBMVR, MVRe).

Parameter		Hazard Ratio	Lower 95%-CI	Upper 95%-CI	p-value
**Group comparisons:**	**Time interval**				
SBMVR vs. SMMVR (ref)	0-2 years	3.948	1.587	9.822	0.003
	2-4 years	0.748	0.203	2.751	0.662
	> 4 years	1.283	0.384	4.283	0.686
SBMVR vs. MVRe (ref)	0-2 years	5.878	3.161	10.932	<0.001
	2-4 years	2.155	0.766	6.064	0.146
	> 4 years	1.674	0.758	3.694	0.202
SMMVR vs. MVRe (ref)	0-2 years	1.489	0.633	3.501	0.362
	2-4 years	2.882	0.936	8.879	0.065
	> 4 years	1.305	0.444	3.833	0.628
**Investigated Confounder:**					
Age at surgery		0.980	0.955	1.006	0.123
Sex: F (ref: M)		0.795	0.476	1.327	0.380
Combined surgery (ref: no)		2.550	1.481	4.390	0.001
Infectious diseases (ref: no)		1.092	0.479	2.487	0.834
Diabetes mellitus (ref: no)		0.798	0.356	1.790	0.585
Adiposity (ref: no)		0.968	0.394	2.381	0.944
Hyperlipidemia (ref: no)		0.887	0.434	1.813	0.742
Hyperuricemia/gout (ref: no)		0.119	0.023	0.616	0.011
Valvular, rhythmological, and other cardiomyopathies (CMPs) (ref: no)		0.760	0.361	1.597	0.469
Atherosclerosis (ref: no)		4.750	1.916	11.774	0.001
Pulmonary disease (ref: no)		0.870	0.312	2.428	0.791
Stomach and duodenal ulcers and inflammation (ref: no)		2.434	0.991	5.976	0.052
Intestinal diseases (ref: no)		1.671	0.432	6.461	0.457
Liver diseases (ref: no)		4.486	1.984	10.143	<0.001
Kidney disease (ref: no)		1.416	0.764	2.625	0.269
Ischemic cardiomyopathies (CMP) (ref: no)		1.483	0.767	2.869	0.242
Malignant diseases (ref: no)		6.128	2.431	15.451	<0.001
**Interaction effects Surgery Type x Age:**					
SBMVR vs. SMMVR (ref)		0.908	0.841	0.980	0.014
SBMVR vs. MVRe (ref)		0.931	0.893	0.971	0.001
SMMVR vs. MVRe (ref)		1.026	0.950	1.108	0.514

Table S24: Hazard ratios (HRs) and corresponding 95% confidence intervals (CIs) from IPTW multivariable cox regression model accounting for all listed confounders for bleeding other than ICH.
